# The kynurenine pathway relates to post‐acute COVID‐19 objective cognitive impairment and PASC

**DOI:** 10.1002/acn3.51825

**Published:** 2023-06-15

**Authors:** Lucette A. Cysique, David Jakabek, Sophia G. Bracken, Yasmin Allen‐Davidian, Benjamin Heng, Sharron Chow, Mona Dehhaghi, Ananda Staats Pires, David R. Darley, Anthony Byrne, Chansavath Phetsouphanh, Anthony Kelleher, Gregory J. Dore, Gail V. Matthews, Gilles J. Guillemin, Bruce J. Brew

**Affiliations:** ^1^ Peter Duncan Neuroscience Research Unit St. Vincent's Centre for Applied Medical Research Darlinghurst New South Wales Australia; ^2^ School of Psychology UNSW Sydney New South Wales Australia; ^3^ Neurology Department St. Vincent's Hospital Darlinghurst New South Wales Australia; ^4^ School of Psychology Macquarie University Sydney New South Wales Australia; ^5^ Macquarie Medical School Macquarie University Sydney New South Wales Australia; ^6^ PANDIS.org Sydney New South Wales Australia; ^7^ Faculty of Medicine UNSW Sydney New South Wales Australia; ^8^ Respiratory Medicine Department St. Vincent's Hospital Darlinghurst New South Wales Australia; ^9^ Kirby Institute UNSW Sydney New South Wales Australia; ^10^ Infectious Disease and Immunology Department St. Vincent's Hospital Darlinghurst New South Wales Australia

## Abstract

**Objective:**

To determine the prevalence and natural history of post‐acute COVID‐19 objective cognitive impairment and function, and their relationship to demographic, clinical factors, post‐acute sequelae of COVID‐19 (PASC), and biomarkers.

**Methods:**

A total of 128 post‐acute COVID‐19 patients (age = 46 ± 15; 42% women, acute disease severity: not hospitalized: 38.6% mild: 0–1 symptoms, 52% 2+ symptoms; 9.4% hospitalized) completed standard cognition, olfaction, and mental health examinations 2‐, 4‐, and 12‐month post diagnosis. Over the same time frame, WHO‐defined PASC was determined. Blood cytokines, peripheral neurobiomarkers, and kynurenine pathway (KP) metabolites were measured. Objective cognitive function was demographically/practice corrected, and impairment prevalence was determined using the evidence‐based Global Deficit Score method to detect at least mild cognitive impairment (GDS > 0.5). Linear mixed effect regression models with time effect (month post diagnosis) evaluated the relationships to cognition.

**Results:**

Across the 12‐month study period, mild to moderate cognitive impairment ranged from 16% to 26%, and 46.5% were impaired at least once. Impairment associated with poorer work capacity (*p* < 0.05), and 2‐month objectively tested anosmia (*p* < 0.05). PASC with (*p* = 0.01) and without disability (*p* < 0.03) associated with acute COVID‐19 severity. KP measures showed prolonged activation (2 to 8 months) (*p* < 0.0001) linked to IFN‐beta in those with PASC. Of the blood analytes, only the KP metabolites (elevated quinolinic acid, 3‐hydroxyanthranilic acid, kynurenine, the kynurenine/tryptophan ratio) associated (*p* < 0.001) with poorer cognitive performance and greater likelihood of impairment. PASC, independent of disability associated with abnormal kynurenine/tryptophan (*p* < 0.03).

**Interpretation:**

The kynurenine pathway relates to post‐acute COVID‐19 objective cognitive impairment and PASC, thereby enabling biomarker and therapeutic possibilities.

## Introduction

There is evidence that SARS‐CoV‐2 is associated with cognitive impairment post‐acute illness^1^, ^2^, ^3^, ^4^, ^5^, ^6^ but the extent, trajectory, and the nature of this new condition are unclear especially in patients recovering from mild to moderate acute infection. Severe cases (those requiring hospitalization) are at risk for both indirect and direct mechanisms of COVID‐19 associated brain damage, particularly from hypoxia, stroke,^1^, ^2^, ^3^, ^4^, ^5^, ^6^ as well as the immune and inflammatory response to SARS‐CoV‐2,^2^ and consequently are at much greater risk of neurocognitive impairment.^5^ Mild to moderate cases of COVID‐19 (not requiring hospitalization) are still at risk of brain dysfunction, and cognitive deficits^1^ but epidemiological longitudinal rigorous studies using objective cognitive testing are in the minority. Similar drawbacks relate to appropriate correction for demographic variables, practice effect on repeated cognitive testing, medical, and psychiatric conditions; see^7^, ^8^ and our review in supplemental material. As most patients with SARS‐CoV‐2 do not require hospitalization, cognitive changes associated with mild to moderate COVID‐19 disease would have significant implications for public health given the current global prevalence of the infection.

Cognitive deficits are a major component of post‐acute sequelae of COVID‐19 (PASC) also known as Long COVID.^9^ PASC definitions concentrate on patients' self‐report symptoms' temporality following the framework of post‐infective fatigue syndrome research.^10^ However, the connection between overall subjective (and cognitive symptoms in particular) with objective cognitive deficits in PASC is complex and not fully understood.^11^, ^12^, ^13^ This is further augmented by data suggesting that PASC has various phenotypes, although there can be overlap.^9^ The time frame of PASC assessment is also important, and research relevant to the current study (Alpha to Delta variants of SARS‐CoV‐2) shows that it is important to consider acute and persistent PASC (occurring 0–30 days post COVID PCR and persisting 30–120 days post‐test) as in the WHO definition, but also late/incident PASC (occurring initially 30–120 days post‐test).^14^

In the current study, our primary aim was to determine the prevalence of post‐acute COVID‐19‐related objective cognitive impairment using an evidence‐based definition of impairment (Global Deficit Score, GDS > 0.5 to detect at least mild cognitive impairment^15^) that was demographically corrected and practice effect‐corrected longitudinally.

Our second aim was to elucidate the natural history and pathogenesis of post‐acute COVID‐19‐related objective cognitive impairment and function by assessing its association with demographic, objectively tested olfaction, mental health, clinical factors, WHO‐informed, self‐report and evidence‐based^14^ PASC, cytokines [interferons (IFN‐beta and GMCSF), major Interleukins, monocyte chemoattractant protein‐1 (MCP‐1), and tumor necrosis factor‐α (TNF‐α)], peripheral neurobiomarkers^16^ (neurofilament light chain (NfL); glial fibrillary acidic protein (GFAP); S100β), and kynurenine pathway (KP) metabolites. We included the plasma KP^17^, ^18^ because of its role in neuroviral‐associated cognitive impairment.^19^, ^20^, ^21^ Indeed, the KP is an IFN stimulated myeloid cell mediated tryptophan degradation pathway important in immune tolerance, neurotoxicity, and vascular injury. Further, the KP is dysregulated in COVID‐19 infection^22^ including in mild cases,^23^ although sex may have a moderating effect.^24^

## Materials and Methods

The study reporting follows the STROBE guidelines for cohort and observational studies.

### Participants

A total of 128 unvaccinated participants with nasopharyngeal swab PCR‐confirmed SARS‐CoV‐2 infection were enrolled through the Sydney St. Vincent's Hospital COVID‐19 ADAPT prospective study March 2020 and July 2021 during the period of SARS‐CoV‐2 variants Alpha to Delta.^25^

### Design

**Figure 1:**
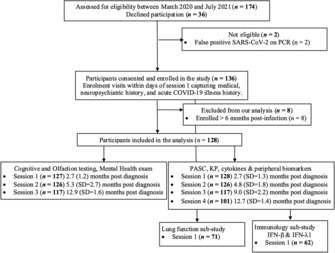
STROBE flow chart, study design. % TLC, % predicted total lung capacity; % DLCO, % predicted hemoglobin‐corrected diffusing capacity of the lung for carbon monoxide. The study is ongoing; the first examination was on May 14, 2020; and the data for the current analyses were censored on September 15, 2021, during the Alpha to Delta wave. The inclusion criteria were being at least 18 years of age, being SARS‐CoV‐2+ on a PCR test. Minor difference in timeline for sessions is due to a few patients having to book their blood tests a few days separately from the neurocognitive assessment because of personal schedule. Based on acute COVID‐19 illness history, the participants were divided into three acute severity groups (mild, moderate, and severe). Detailed information for this classification has been previously reported in Darley et al.^25^ Mild Managed in the community; minor, largely upper respiratory tract viral symptoms, including sore throat, rhinorrhea, headache, and anosmia/ageusia. Moderate Managed in the community with fever/chills AND one of the following organ‐localizing symptoms, OR at least two of the following organ‐localizing symptoms: cough, hemoptysis, shortness of breath, chest pain, nausea/vomiting, diarrhea, altered consciousness/confusion. Severe required inpatient care (wards or intensive care unit). The cohort at 2‐month post diagnosis was composed of 39% with mild disease (*n* = 50), 52% with moderate. PASC refers to the PASC related self‐report data collection and assessments detailed in the methods and supplementary material.

The study is a prospective observational study with two main longitudinal components: (1) the neurocognitive assessment, and (2) the clinical and blood test visit assessment (Fig. 1: STROBE flow chart).

### Procedures

#### Standard cognitive testing

The cognitive test selection was informed by the International Neuropsychology COVID‐19 taskforce^26^ to target cognitive functions affected in COVID‐19. The CogState Computerized Battery (CCB) is a widely used and validated cognitive screening test that has been specifically designed for repeated testing in longitudinal (test–retest reliability *r* = 0.8).^27^ The CCB data were normalized for demographic effects (age, education, and sex) and corrected for practice effect.^27^ The CCB demographically corrected individual task *z*‐scores were averaged into a mean *z*‐score. A higher *z*‐score represents a better objective global performance. Next, the five individual *z*‐scores were transformed into deficit scores ranging from 0 to 5. The deficit scores were subsequently averaged into a Global Deficit Score (GDS) where a higher GDS indicated greater impairment. We used best‐evidence clinical cutoff (GDS > 0.5) to classify at least mild objective cognitive impairment.^17^ The same method classifies 5% of healthy controls with comparable demographics as impaired.^15^, ^27^

#### Objective olfaction, self‐reported everyday function, and standard mental health assessments

These assessments have been described in Ref. [25] (see also supplemental material).

#### Medical visit, blood draw and lung function and acute disease severity

As part of the ADAPT protocol,^25^ patients underwent a demographic and medical history inventory 2‐month post diagnosis to determine acute disease severity based on 18 symptoms and hospitalization status (see Table S1, 38.6% mild: 0–1 symptoms not hospitalized, 52% 2+ symptoms not hospitalized, 9.4% hospitalized). A subset enrolled into the lung function sub‐study. For the current analyses, we selected the two outcomes that were most related to disease severity as shown in Ref. [25]: at 2‐month post diagnosis % predicted total lung capacity (TLC) and % predicted hemoglobin‐corrected diffusing capacity of the lung for carbon monoxide (DLCO) (see also supplemental material).

#### PASC definition (see also supplemental material)

PASC was based on 18 symptoms collected at 2, 4, 8, and 12 months, and used the WHO definition partially modified to include incident late period PASC as defined by Ref. [14]. Following a post‐infective fatigue syndrome research framework,^10^ the participants also completed the Visual Analogue Fatigue Scale, and a disability scale. Self‐reported cognitive symptoms were assessed with the Patient Assessment of Own's Functioning Inventory (PAOFI)^12^ at 2‐month and at least one more time across the study period for participants with follow‐up. We classified participants as having PASC (*n* = 70, 55%) or not having PASC (*n* = 57, 45%). We also classified participants as having PASC “only” (*n* = 33, 26%), PASC with disability (*n* = 37, 29%), or not having PASC (*n* = 57, 45%).

#### Quantification of biomarkers

Detailed methods are presented in supplemental material. The KP was quantified using Ultra High‐Performance Liquid Chromatography (uHPLC) as previously described.^28^ IFN‐β were quantified via LEGENDplex Human Anti‐Virus Response Panel as previously described.^29^ The quantification of the cytokines and chemokines in the serum was carried out by Eve Technologies (Calgary, Canada) using the human focused 15‐Plex Discovery Assay. Peripheral neurobiomarkers were quantified using standard enzyme‐linked immunosorbent assays.

### Statistical analyses

Data formatting, transformation, and missing data analysis, covariates selection and analyses, cognitive definitions for the cross‐sectional and longitudinal cognitive analyses are presented in supplemental material.

Cross‐sectionally, we compared cases with cognitive impairment and those without on all relevant outcomes at the initial 2‐month visit using chi‐square, ANOVA/*t*‐tests as appropriate. Cognitive impairment in the COVID‐19 sample was compared to pre‐COVID norms^27^ impairment rate (5%) on the CBB in a demographically comparable sample of *N* = 119 using chi‐square. To assess any association between functional status and cognitive impairment, we compared the participants classified as having cognitive impairment versus those without using chi‐square. Finally, cognitive impairment was also defined as being impaired at least once across the 12‐month study period.

Longitudinally, we used a series of multilevel models (i.e., general linear mixed models, see detailed rationale in supplemental material). First, time as months post‐infection was entered as a fixed continuous predictor. Second, to account for the initial cognitive performance,^30^ we used the 2 months post diagnosis cognitive impairment status (initial visit impaired vs. unimpaired status) as a fixed effect predictor (main effect) and its interaction with time (slope).

#### KP profile and predictor analyses and covariates

The KP longitudinal dynamics were tested in each metabolite separately and for the KYN/TRP ratio in analyses where the KYN had a significant effect. Using the best fit mixed effect models, we tested all covariates including PASC, and sex (fixed effect and time interaction) to plan for the subsequent analyses.

#### KP and cognition longitudinal models and covariates

We used a series of multilevel models (i.e., general linear mixed models) to test how the KP contributed to cognitive function (main effect) and time interaction (slope) following the analytic steps recommended by Hoffman.^31^ Each KP metabolite was entered as a fixed continuous predictor (main effect), along with time (month post‐infection), and KP metabolites interaction with continuous time (slope) and relevant covariates that were found to be significant in the previous analyses as fixed effect and three‐way interaction (PASC). To better understand the impact of the KP on impairment across the study period, we also tested the QUIN age‐norm cutoff on impairment over time defined as a dummy variable to enable the inclusion of a random subject effect.

#### KP and IFN‐β analyses: healthy control and other coronaviruses data^29^ on the KP

Using a sub‐sample (*n* = 62) of the current cohort which had IFN‐β measured concomitantly to the 2‐month post diagnosis cognitive session, we determined their association with the KP metabolites^29^ with a mixed effect model (subject as a random effect, PASC (*n* = 31) vs. no PASC (*N* = 31) as a fixed main effect, the IFN as a fixed main effect and its interaction). The same control data in a subset (healthy control *N* = 25 and other coronaviruses *N* = 25) were also assayed for the KP using the same methods as described above and compared with the current COVID‐19 sample.

#### KP, other cytokines, and peripheral brain biomarkers

All biomarkers were Log transformed except for MCP‐1 which was normally distributed. We conducted final mixed effect models using two of the KP metabolites (top and low sections of the KP: √KYN and Log QUIN) that were significantly associated with cognition in the main model's analyses as outcomes. We also tested the biomarkers directly against cognition. For each model, we tested main effects and time interaction effects as in previous models.

#### Power and experiment‐wise corrections, and software.

Considering the exploratory nature of the analyses and the need to control for experiment‐wise comparisons' error rate, we used false‐discovery rate (FDR) correction^32^ within each main model as well as use random variable selection to reduce the number of covariates added to the main model. Using this strategy, our main model had 100% power for the main effects and 65%–80%% power for a time interaction while also including a covariate (unconditional Hotelling Lawley Trace, unconditional, *p* < 0.05) to detect a small to medium effect size for KP and cognition time association. Power computations were conducted in GLIMMPSE.^33^ Moreover, effect sizes were extracted to assist in the interpretation of the significance of the effects. All *p*‐values are two‐tailed. Exploratory analysis *p*‐value was set at *p* < 0.05. Analyses were conducted in SPSS V.28 and JMP V.15 (SAS Inc).

## Results

The 2 months post‐acute infection demographic and clinical characteristics of the total study cohort including comparison of those with and without objective cognitive impairment are presented in Table 1. Cognitive impairment was associated with objectively tested anosmia (*p* = 0.05), poorer work capacity (*p* = 0.03), and elevated QUIN and KYN/TRP ratio. There was only a modest association with PASC when considering the effect size (*d* = 0.80).

**Table 1:** Demographic and clinical characteristics at 2 months post diagnosis in the entire sample and by objective cognitive impairment status.

Sample characteristics	Full sample (*n* = 127)		Impaired (*n* = 20)		Unimpaired (*n* = 107)		*p*	*d* ^a^
	*M*	SD	*M*	SD	*M*	SD		
Age (years)	46.7	15.0	47.9	16.2	46.4	14.8	0.67	0.10
Educations (years)	15.3	2.8	14.0	3.1	15.5	2.7	0.04	0.50
Mental health composite	0.1	1.6	0.5	2.1	0.0	1.5	0.24	0.30
DMI‐10	6.1	6.6	7.3	7.9	6.0	6.3	0.39	0.20
IES‐R	12.8	14.1	17.3	18.8	12.0	12.9	0.12	0.37
SPHERE‐Psych	1.9	2.6	2.2	3.1	1.8	2.5	0.46	0.15

	*n*	%	*n*	%	*n*	%	*p*	*d* ^a^
Sex (female)	53	41.7	8	40	45	42	0.86	0.04
ESB^b^	115	90.5	4	20	8	7.5	0.8	0.60
Medical comorbidities^c^	46	36.2	7	35	39	36.45	0.90	0.03
Prior psychiatric diagnosis	11	8.7	3	15	8	7.5	0.30	0.43
DMI‐10 ≥ 9	20	15.7	7	35	28	26.2	0.42	0.23
IES‐R ≥ 33	15	11.8	3	15	12	11.2	0.63	0.18
SPHERE‐Psych ≥ 2	49	38.6	8	40	41	38.3	0.89	
COVID‐19 disease severity
Mild (ref)	49	38.6	9	45	40	37.4	0.68^f^	0.04
Moderate	66	52	10	50	56	52.3	0.64	0.05
Severe	12	9.4	1	5	11	10.3	0.40	0.43
PASC%	70	55.1	14	70	56	52.3	0.15	0.80
PASC “only”	33	25.98	7	35	26	24.3	0.33	0.28
PASC with disability%	37	29.1	7	35	30	28.04	0.33	0.18
%TLC < 90^d^	16	20.0	1	12.5	15	20.8	0.57	0.33
% DLCO > 90^d^	37	46.2	3	37.5	34	47.2	0.60	0.22
2‐month impaired olfaction^e^	126	24.6	19	42.1	107	21.5	0.05	0.54
Self‐reported acute anosmia	127	57.5	10	50	63	58.9	0.46	0.18
Self‐reported dysgeusia	126	55	11	55	44	41.1	0.48	0.31
Self‐reported myalgia	127	53.5	10	50	58	54.2	0.72	0.09
Work efficiency^g^	10	9	4	22.2	6	6.5	<0.04	0.78
QUIN >650 nM (% elevated)	51	41.1	14	73.7	37	35.2	<0.002	0.90
KYN/TRP 10*3 > 63 (% elevated)	47	37.9	12	63.2	35	33.3	<0.02	0.66
QUIN & KYN/TRP (% elevated)	36	28.3	12	60	24	22.4	0.0006	0.91

Ref: reference, mild acute disease severity was compared to moderate and severe acute disease severity. *Mild acute disease* patients were community‐managed with minor, mostly upper respiratory symptoms. *Moderate acute disease* patients were community‐managed with fever or chills and experienced at least one of the following symptoms: cough, shortness of breath, chest pain, nausea or vomiting, diarrhea, mild instances of altered consciousness or confusion, abnormal lung function. Patients were also classified as moderate illness if they did not experience fever or chills and presented with at least two of the aforementioned symptoms. *Severe acute disease*: Patients who had been hospitalized (see Ref. [25] for more details). ns, not significant at *p* < 0.05.

^a^ Cohen's *d* with Hedges correction for sample *N* ≤ 20.

^b^ ESB: English speaking background assessing geographical background/race/ethnicity. People of non‐ESB included Asian Australians born in Australia for the majority, and then South Americans, non‐English speaking European people; all were fluent in English.

^c^ Medical comorbidities which were investigated/included: chronic cardiac disease (*n* = 10); hypertension (treated) (*n* = 16); obesity (*n* = 7); chronic lung disease (*n* = 1); asthma (treated) (*n* = 11); diabetes (treated) (*n* = 8); chronic kidney disease (*n* = 3); liver disease (*n* = 0); cancer (treated) (*n* = 7); HIV infection (treated) (*n* = 1); transplant recipient (*n* = 1); rheumatologic or autoimmune disorder (*n* = 9); obstructive sleep apnea (treated but 1), (*n* = 3); other comorbidities (*n* = 2: 1 early Parkinson's disease treated). Neurological assessment was performed on all patients who were identified with cognitive impairment, and additional blood tests and standard MRI brain scans were performed with negative results.

^d^ *n* = 80 for the lung function sub‐study % TLC, % predicted total lung capacity; % DLCO, % predicted hemoglobin‐corrected diffusing capacity of the lung for carbon monoxide. Four women were pregnant but in the gestation period; 8.7% (*n* = 11) has a history of a psychiatric diagnosis (anxiety disorder and major depressive disorder on treatment).

^e^ Impaired olfaction objectively tested on the NIH Toolbox Odor Identification Test. DMI‐10: Depression in the Medical ill scale 10 items; Impact of Event Scale Revised (IES‐R); Somatic and Psychologic Health Report (SPHERE‐34) anxiety‐depression subscale.

^f^ Overall chi‐square *p*‐value.

^g^ Work efficiency at 2 and 4 months. Results were identical. A selection of self‐reported symptoms during acute infection are provided in Table 1; other symptoms are included in supplemental material by disease severity. Note that none of the acute symptoms differed by impairment status. PASC used the WHO definition and incident PASC definition according to Ref. [14], and operationalized following post‐infective research framework as described in Ref. [10].

### Cognitive impairment and cognitive function across the study period (Fig. 2) and covariates

**Figure 2:**
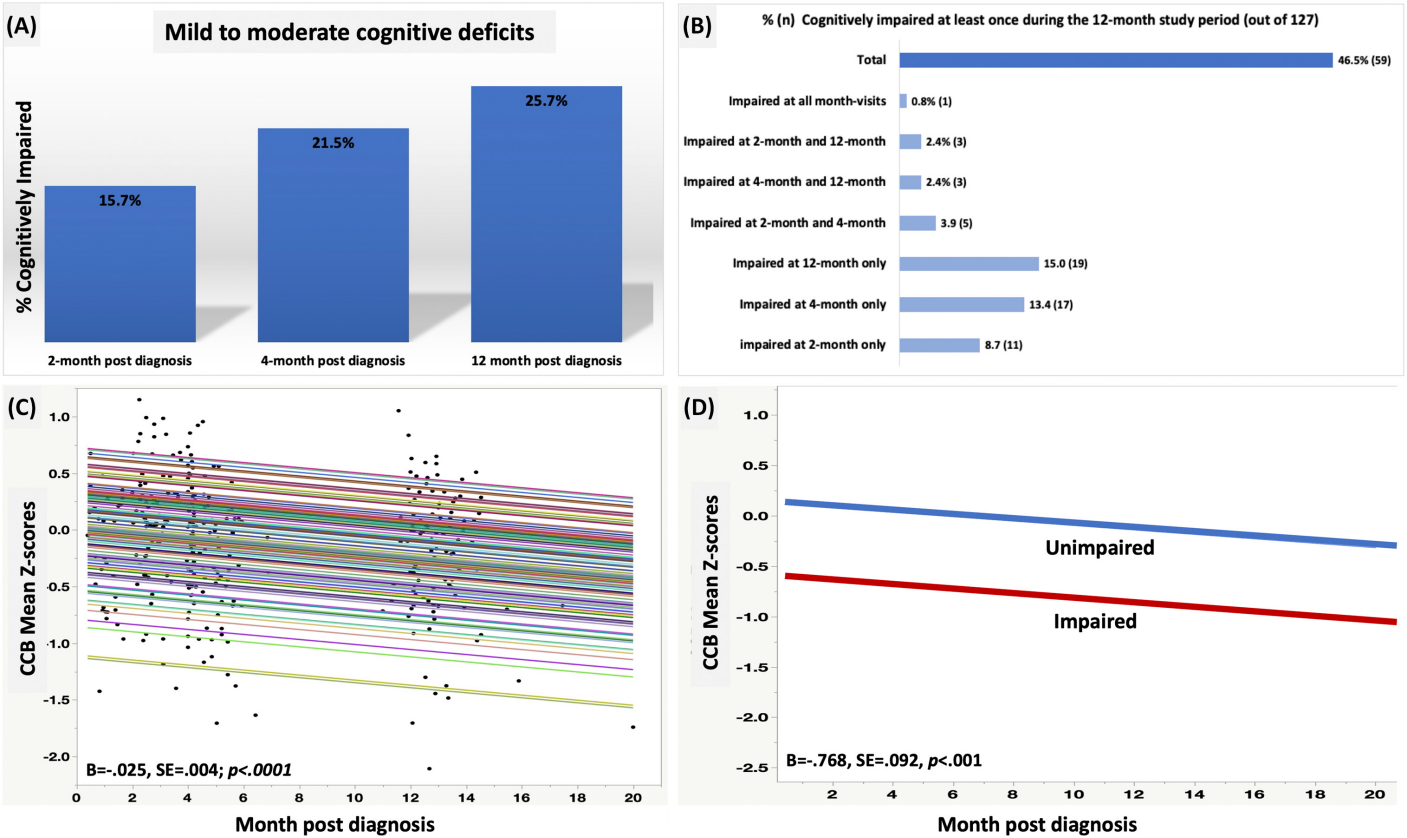
Cognitive impairment and overall cognitive performance and decline 12‐month post‐acute COVID. Deficits were mild to moderate, and no patients had severe cognitive impairment. The best fit model between cognition and the KP was based on a fixed linear time model, with a random intercept. (A) Across assessments, those with at least mildly impaired cognition (GDS > 0.5) increased, although chi‐square test indicated that the proportion of cognitive impairment did not differ significantly at 2, 4, and 12 months, *X* ^2^(2, *N* = 349) = 3.52, *p* = 0.17, *W* = 0.10. (B) Cognitive impairment defined as at least impaired once during the 12‐month study visit. (C) Best fit LME model (Model ‐2LL = 432; AICc = 440) with random intercept (*p* = 0.003), there is a −0.43 of a *z*‐score decline in average [95% CI = −039 to −0.47]. (D) LME (Model ‐2LL = 387; AICc = 395) with the addition of initial visit cognitive impairment status and its interaction with time.

Cognitive impairment was significantly different from the normative reference at all time points (15.7%, *p* = 0.01 at 2 months; 21.5%, *p* = 0.004 at 4 months, and 25.7% *p* < 0.0001 at 12 months). Cognitive performance declined over time by an average slope of 0.43 [95% CI = −0.39 to −0.47] of a *z*‐score. In the model where the initial visit cognitive impairment status was added, months post diagnosis (*p* < 0.001) and impairment status (*p* < 0.001) were significant predictors of cognitive function (main effect), but not their interaction (*p* = 0.64, no time effect on slope). 59/127 (46.5%) were impaired at least once across the study period. Covariates selection showed that only pre‐existing psychiatric conditions were a significant (*p* < 0.03) predictor of cognitive impairment. However, when added to the best fit longitudinal cognitive model, its main effect (*p* > 0.19) or time interaction predictor (*p* > 0.23) was not significant.

### PASC and self‐reported cognitive symptoms

PASC (*p* < 0.03) and PASC with disability (*p* = 0.01) associated with greater acute symptoms severity. There was no difference for, age, education, or sex. Elevated self‐reported cognitive symptoms on the PAOFI (total PAOFI >4) were greater in participants with PASC with disability (54%), than those with PASC only (24.5%), while those without PASC did not report elevated cognitive complaints (by design). On their own, self‐reported cognitive symptoms were not associated with cognitive impairment but were associated with objectively tested olfaction impairment particularly at 12 months (*p* = 0.005). Self‐reported cognitive symptoms were also associated with greater mental health symptoms (*p* = 0.02). Note that self‐reported anosmia was not associated with objectively tested anosmia (*p* > 0.40).

### KP original, transformed, and normed values show a prolonged activation (Fig. 3)

**Figure 3:**
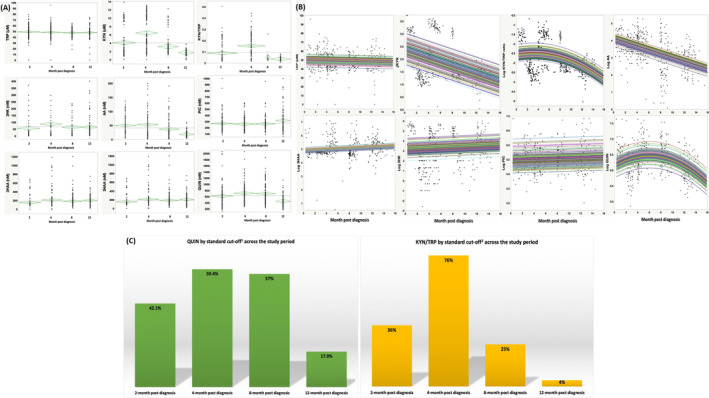
KP dynamics over the study period. (A) Original values with mean and SD and Grand mean diamonds. (B) Transformed values and regression models. Mixed effect model with random subject intercept. Log QUIN and KYN/TRP showed non‐linear (quadratic) time effect. Log 3HK, Log PIC and TRP show no time effect. More specifically, both √KYN and KYN as dummy code, (KYN > 3 μM = 1 vs. 0) decreased over time (√KYN: *B* = −0.086, SE = 0.007, *p* < 0.001; Model ‐2LL = 887, AICc = 897; KYN dummy coded: *B* = −0.47, SE = 0.005, *p* < 0.001; Model ‐2LL = 565, AICc = 574). Log AA decreased over time in a linear pattern at the group level (*B* = −0.107, SE = 0.007, *p* < 0.001; Model ‐2LL = 998, AICc = 1008). Log 3HAA increased over time in a linear pattern at the group level (*B* = 0.24, SE = 0.007, *p* < 0.001; Model ‐2LL = 847, AICc = 857). Log QUIN increased in a quadratic pattern both at the group level (significant fixed quadratic time slope, *B* = −0.13, SE = 0.002, *p* < 0.001), and at the individual level (significant random quadratic time slope *p* < 0.001 at each visit) (Model ‐2LL = 498, AICc = 512). The KYN/TRP model also showed a quadratic pattern driven by random (individual) effects (both intercept and slope, *p* < 0.04) (Model ‐2LL = 779, AICc = 789). Note that only the √KYN (as opposed to KYN as dummy coded) is illustrated as it also presents the individual values in a more realistic fashion that when using the dummy code. Note, however, that the dummy coded outcome model (0 = within normal range; 1 = KYN > 3 μM) showed the best fit (AIc = 574 vs. 897), but same direction of effect. (C) Age‐normed values. The cutoffs were derived from samples that have the same age as the current sample. ^a^QUIN > 605 (nM): reference [34]. The continuous outcomes provide the best model (panel B). ^b^KYN (μM)/TRP (μM) ratio*10^3^ (standard cutoff >63): reference [35].

KP original values are presented in Fig. 3A. Longitudinal analyses using transformed data are presented in Fig. 3B. KYN, KYN/TRP, AA decreased over time, while 3HAA and QUIN increased over time. KYN/TRP and QUIN showed a quadratric pattern of activation. Figure 3C presents the age‐norms data QUIN (>605 nM that is 2 SD > age‐norms in Ref. [34]) and the easiest products to obtain clinically KYN (μM)/TRP (μM) ratio*10^3^ (standard cutoff >63).^35^ At 2 months and independent of disability, an abnormally elevated KYN/TRP ratio was more frequent (*p* < 0.03) in cases with PASC (46.4%) versus those without (27.3%).

### KP covariates

We found a main effect of sex where men had higher Log AA compared to women (*B* = 0.18, SE = 0.087, *p* = 0.04) overall, and with greater decrease across time compared to women (*B* = −0.29, SE = 0.014, *p* = 0.04; Model ‐2LL = 631, AICc = 741). Men also had higher Log PIC (*B* = 0.126, SE = 0.06, *p* = 0.04; Model ‐2LL = 310, AICc = 320). Finally, men had higher Log QUIN compared to women (*B* = 0.181, SE = 0.058, *p* = 0.002; Model ‐2LL = 369, AICc = 379). There was no sex effect for other metabolites. In the models testing the KP and cognition association, sex had no effect. Independent of disability, PASC was associated with an abnormal KYN/TRP ratio across the study period (*B* = 0.09, SE = 0.04, *p* < 0.03; Model ‐2LL = 790, AICc = 802).

### KP, continuous cognitive function over time, and covariates (Table 2)

**Table 2:** Cognition and KP models organized by effect size (medium to small), confidence of interval on the outcome, and *p*‐value.

KP metabolites	Random intercept	Main time effect	Main metabolite effect	Interaction	‐2LL	AICc	Mean *z*‐score slope 95% CI^b^
	*B* (SE) *p*	*B* (SE) *p*	*B* (SE) *p*	*B* (SE) *p*			
√KYN^a^	0.138 (0.065) 0.035	−0.034 (0.014) 0.01	−0.137 (0.033) <0.001	0.000 (0.010) 0.96	378	386	−0.49 [−0.47, −0.51]
Log QUIN	0.66 (0.34) 0.05	−0.12 (0.06) 0.06	−0.123 (0.053) 0.02	0.015 (0.01) 0.15	397	405	−0.38 [−0.37, −0.39]
Log 3HAA	0.360 (0.176) 0.041	−0.003 (0.042) 0.94	−0.094 (0.034) 0.006	−0.004 (0.008) 0.66	398	406	−0.64 [−0.43, −0.79]
Log 3HK	−0.031 (0.061) 0.61	−0.045 (0.013) <0.001	−0.027 (0.014) 0.049	0.007 (0.004) 0.06	395	402	−0.26 [−0.18, −0.33]
TRP	0.032 (0.11) 0.77	0.009 (0.021) 0.65	−0.003 (0.002) 0.14	−0.001 (0.000) 0.10	413	421	−0.32 [−0.31, −0.32]
Log PIC	0.133 (0.34) 0.69	0.042 (0.061) 0.40	−0.045 (0.61) 0.46	−0.012 (0.009) 0.19	402	410	−0.11 [−0.08, −0.14]
Log AA	−0.055 (0.134) 0.68	−0.034 (0.022) 0.12	−0.017 (0.037) 0.65	0.003 (0.007) 0.70	406	414	−0.11 [−0.02, −0.20]

A *z*‐score has a mean of 0 and a SD of 1. FDR across all fixed effects (time, main cytokine, and interaction): *p* ≤ 0.02.

^a^ Note that when using the dummy coded KYN (KYN > 3 μM = 1 vs. 0), results are as follow: Random intercept: −0.41 (SE = 0.44), *p* = 0.36; Main time effect: −0.33 (SE = 0.004), *p* < 0.001, Main metabolite effect: −0.15 (SE = 0.054), *p* = 0.005; Interaction: 0.18 (SE = 0.16), *p* = 0.25, with mean *z*‐score decline 95% CI = 0.17 [0.15–0.19].

^b^ These values are obtained while maintaining month post‐infection centered at 0 (i.e., the sample's mean).

We found a significant negative association between KYN (and KYN/TRP), 3HK, 3HAA, and QUIN (FDR significant) and cognitive function (main effect). However, there was no time interaction effect between the KP and cognition (KP was not associated with slope of cognitive function which was shown to decline in the cognitive models presented above). There were no other significant effects for any of the tested clinical factors or biomarkers.

### KP, IFN‐β, and other biomarkers; KP, IFN‐β, PASC, and 2 months objective cognitive impairment

**Figure 4:**
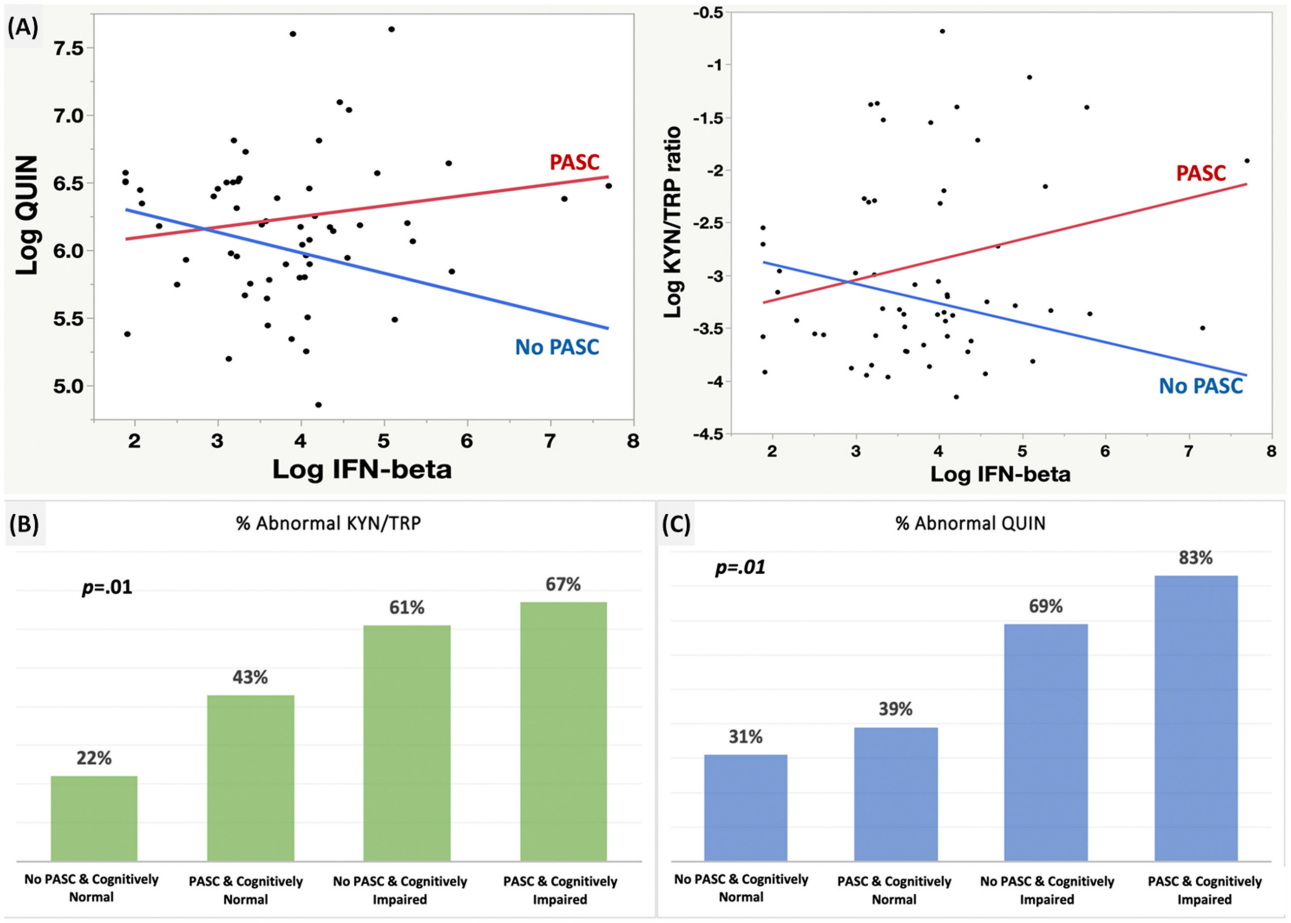
KP, IFN‐β, PASC, and 2‐month objective cognitive impairment. (A) Sub‐sample of 62 COVID‐19 cases from the same cohort, with half with PASC and half without. IFN‐β was associated with Log QUIN in those with PASC (*B* = 0.11, SE = 0.07, *p* = 0.10; significant FDR corrected). IFN‐β is associated with Log KYN/TRP in those with PASC (*B* = 0.19, SE = 0.11, *p* = 0.09; significant FDR corrected). IFN‐β was not directly associated with cognition. (B) PASC, 2‐month objective cognitive impairment and QUIN. Overall chi‐square = 11, *p* = 0.01, and is driven by the effect of cognitive impairment. (C) PASC, 2‐month objective cognitive impairment and KYN/TRP. Overall chi‐square = 10.7, *p* = 0.01, and is driven by the effect of PASC.

The KP was associated with IFN‐β at 2 months post diagnosis (Fig. 4A). No blood analytes besides the KP were directly associated with cognition (see supplemental material for the results with the peripheral neurobiomarkers, note that QUIN showed a positive significant association (FDR‐corrected, *p* < 0.001) with IL‐6). Figure 4B shows that objective cognitive impairment with PASC at 2 months post diagnosis was associated with the highest QUIN elevation, although those without PASC and cognitive impairment also had elevated QUIN. Figure 4C illustrates that an abnormal KYN/TRP was more dependent on PASC and objective cognitive impairment at 2 months post diagnosis.

### KP, dichotomous cognitive impairment at 2 months, and over the study period, and PASC

**Figure 5:**
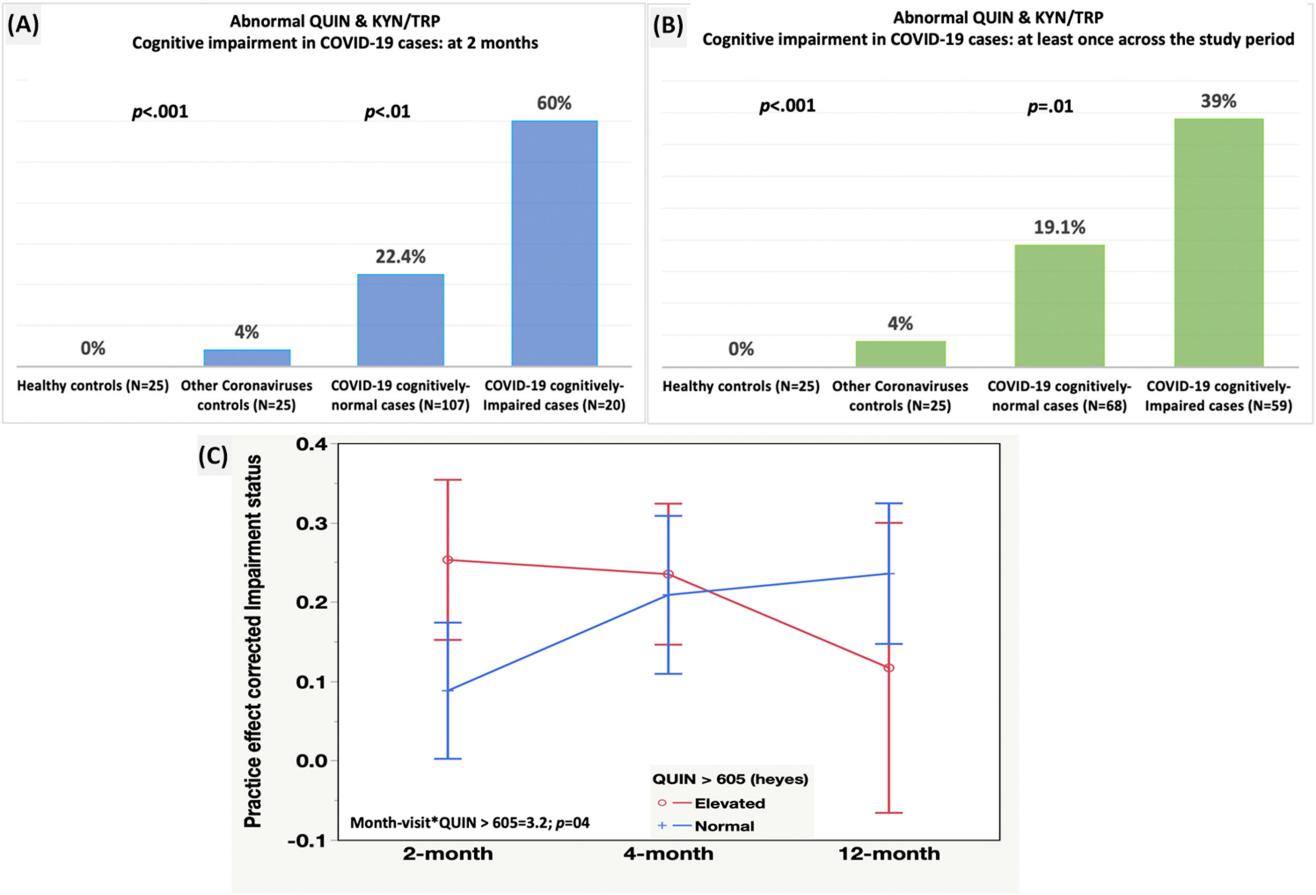
QUIN and KYN/TRP cutoffs in the COVID‐19 sample versus published control data and effect on cognitive impairment over time. (A) Results considering cognitive impairment status at 2 months only. Multiple comparisons use the COVID‐19 impaired cases as the comparator. (B) Results considering cognitive impairment status as impaired at least once across the study period. Multiple comparisons use the COVID‐19 impaired cases as the comparator. (C) Elevated QUIN predict greater chance of cognitive impairment over time and pattern of cognitive impairment over the study period. Details on the healthy controls and other coronaviruses data who were also enrolled into the ADAPT are published in Ref. [29]. Elevated QUIN (>605 nmol) and elevated KYN/TRP 10.3 >63. Cognitive impairment is defined as a dummy variable (1/0) to enable the use of a random subject effect (Model ‐2LL = 290.8, *p* < 0.001). In all instance, the cognitively impaired COVID‐19 cases were more likely to have an abnormal QUIN and KYN/TRP ratio. Note that the cognitive data were MCAR.

Using previously published control data^29^ in healthy individuals and coronaviruses controls, the KP activation observed in the COVID‐19 sample has a unique pattern compared to the other groups at 2 months (Fig. 5A). The KP association with objective cognitive impairment was also evident when impairment was defined as being impaired at least once across the study period (Fig. 5B) (*N* = 59). In the model inspecting QUIN as an age‐norm cutoff (>605 nmol) and the impairment over time as a dummy variable, we found that elevated QUIN predicted greater likelihood of cognitive impairment over the study period (Fig. 5C).

## Discussion

Our study determined the prevalence and investigated the natural history and pathogenesis of post‐acute COVID‐19‐related objective cognitive impairment and function in SARS‐CoV‐2 PCR‐confirmed unvaccinated patients following predominantly mild to moderate acute COVID‐19 disease.

Mild to moderate cognitive impairment was present in 16%–26% participants *across* the study period with 46.5% having impairment at one or more time points during the study period. While no patient demonstrated severe cognitive impairment, this prevalence rate was greater than pre‐COVID normative references. Despite detecting only mild to moderate cognitive deficits, cognitive impairment at 2 and 4 months was associated with a lower capacity for returning to pre‐COVID work. In cross‐sectional analyses, cognitive impairment at the 2‐month initial visit was associated with impaired objectively tested olfaction, an activated KP (i.e., QUIN and KYN/TRP) and PASC to a lesser extent. Across the 12‐month study period, cognitive function showed a modest declining slope and the magnitude of this decline did not differ between those with or without cognitive impairment at the initial visit.

Across the 12‐month period, the KP, namely QUIN, 3HAA, and KYN (and KYN/TRP), showed a prolonged (2 to 8 months) activation compared to age‐control reference data in a (quadratic) pattern with recovery for most between 8 and 12 months (17.9% still had elevated QUIN at 12 months).

The pattern of activation of the KP was associated with poorer cognitive function, and greater likelihood of cognitive impairment over‐time. Importantly, no other blood biomarkers, sex, or clinical factors (pre‐existing mental health or mental health during the study period, olfaction, medical comorbidities, disease severity or respiratory function) were associated with cognition.

Finally, while PASC associated with an abnormal KYN/TRP ratio, the KP association with objective cognitive impairment and function was the strongest and most robust. Interestingly, the KYN/TRP ratio association with PASC was independent of disability. Both QUIN and KYN/TRP were mediated by IFN‐beta in those with PASC corroborating and extending earlier findings.^29^

Compared to the existing literature,^6^, ^7^, ^8^, ^36^, ^37^, ^38^, ^39^ our study is the only study which provides clear prevalence figures across a 12‐month period of post‐acute COVID‐19 objective cognitive impairment adjusted for multiple confounds including demographics and practice effect. Practice effect correction provides more accurate prevalence rate at follow‐up and across the study period.^30^ The few existing studies that have used well validated objective testing, and appropriate correction for demographics to determine cognitive impairment (also using pre‐COVID norms) show mostly mild to moderate cognitive deficits like our study. While the degree of cognitive impairment was not high, it was significant enough to impact self‐report capacity of returning to work, but not everyday function and exercise. This result may inform more refined PASC definitions because it shows that not all forms of COVID‐19 related disability are affected by mild to moderate objective cognitive impairment, but probably only the most demanding.

Objectively tested olfaction, and objective cognitive impairment were only associated at the 2 months suggesting that both conditions do not systematically co‐evolve. Importantly, objectively tested olfaction and self‐reported anosmia were never significantly linked across the study period corroborating evidence from olfaction research that self‐report is unreliable.^40^ This result should also inform more accurate PASC definitions where objective olfaction testing is required.

The lack of relationship between cognitive impairment and systemic disease severity or respiratory function should be interpreted with caution. The subjects in our study had predominantly mild to moderate acute COVID and the few that were hospitalized did not die. Nonetheless, several other studies have found similar results.^3^, ^7^, ^8^ This finding has major public health and economic implications: relying on disease severity (especially mild to moderate distinction) to estimate disease burden would result in a significant underestimate.

PASC showed only a modest association with objective cognitive impairment and only at 2 months. Furthermore, the subjective cognitive symptom component of PASC was not associated with objective cognitive impairment but was more strongly associated with objectively tested olfaction impairment and mental health symptoms. These results are partly in line with our research in NeuroHIV which shows that self‐report tends to be strongly associated with other forms of self‐report.^11^ The link between objective anosmia and cognitive symptoms further shows the importance of olfaction deficits in the natural course of the infection and is explained by the fact that those with persistent anosmia have poorer outcomes (data not shown). Overall, the relationship between self‐report of cognitive symptoms and objective cognitive impairment is complex,^11^, ^12^, ^13^ especially in the context of a dynamic post‐viral syndrome which can result in various phenotypes^41^ likely underlined by different etiologies.^9^ Further, cognitive fatigue post‐viral illness fluctuates and may be more evident after a stronger/longer cognitive effort^42^ than what may be required during a short screen, although the CBB accurately detect mild cognitive deficits/change.^27^ The affective component of PASC associated cognitive symptoms^13^ warrant further study because this profile often predicts cognitive decline in the longer‐term.^43^

Overall cognitive performance modestly declined across the 12‐month study period at the group level with a small to medium effect size decline (slope of −0.43 of a *z*‐score on average). The magnitude of this cognitive decline was independent from the initial cognitive impairment status implying an underlying pathogenic mechanism across the sample which is a novel finding. This contrasts with studies which have focused on severe cases of acute COVID‐19 where pre‐morbid neurological conditions predict cognitive deterioration.^5^ Our finding of cognitive decline is in accordance with cognitive^44^ and brain imaging^45^ studies at 12 months post diagnosis. Studies with longer follow‐up will be critical to determine whether this trend is maintained.^46^

Based on our review and that of others,^7^, ^8^ cognitive studies have not concomitantly assessed targeted biomarkers. Neurobiomarker studies^2^, ^16^ (NfL; GFAP; S100β; GMCSF) have noted abnormalities but these have been in the acute infection stage often with severe disease. To the best of our knowledge,^9^ our finding of an association between the prolonged (2 to 8 months) activation of the KP (itself IFN‐beta/IL6 mediated and shown in our study) and poorer cognitive function and cognitive impairment over time is unique. Furthermore, the combination of elevated KYN/TRP (easy to measure in clinical practice) and QUIN was associated with cognitive impairment when defined as at least impaired once across the study period. This effect is further corroborated when elevated QUIN is shown to predict greater chance of impairment over time.

PASC, independent of disability and cognitive impairment mostly, was associated with an abnormal KYN/TRP ratio confirming that the KP is involved in PASC.^22^, ^23^ While most PASC phenotypes have an underlying inflammatory nature,^9^, ^22^, ^23^, ^29^ (evident in this study when we show that those with PASC are more likely to have both an activated KP and elevated IFN‐beta) cognitive impairment appears to be dependent on the KP neurotoxic pathway: QUIN. This in turn suggests that PASC has organ or phenotype specific pathogeneses further implying the need for a combination of biomarkers and treatments. Finally, our result suggests that PASC without obvious disability is also important to detect.

There is biological plausibility for a role of the KP in the pathogenesis of post‐acute COVID‐19 objective cognitive impairment and function. QUIN is a known neurotoxin produced by monocyte–macrophage lineage cells under regulation by cytokines especially the interferons and IL6.^17^, ^18^, ^22^, ^47^ Furthermore, quinolinate phosphoribosyltransferase, the enzyme in the KP responsible for quinolinic acid production, is easily saturable at low concentrations of QUIN while the other KP enzymes are not. When the KP is activated, shunting of KP products through the pathway will be “banked up” especially QUIN. If there is continued replenishment of tryptophan as occurs in the normal diet, tryptophan concentrations will be in the normal range yet KP products especially QUIN will be elevated.^17^, ^18^, ^47^ This is the likely explanation for the lack of variation in tryptophan in our study despite KP activation. There are further data to support a pathogenetic role for the KP: SARS‐Cov‐2 can efficiently infect macrophages but leads to an abortive infection,^48^ and macrophages are a prominent feature of the neuropathology of COVID‐19.^2^ Furthermore, IFN‐β^29^ and IL‐6^2^ are markers of PASC and were associated in our study with KP activation. Some factors that likely modulate the involvement of the IFN‐mediated KP, or that are directly involved in the cognitive decline that was observed and not measured in the current study include adaptive immunity, and host genetic factors which have been shown to play a role in COVID‐19 disease severity (e.g., autoantibodies against type I IFNs^49^).

Existing studies and our results allow a testable hypothetical model to be considered. KP activation occurs through cytokines especially IFN‐β and IL‐6.^47^ This would normally down regulate immune activation to tolerance, but the SARS‐CoV‐2 infection of monocytes and macrophages (and possibly microglia) maintains KP activation through the latter cytokines.^18^, ^47^, ^48^ This would allow persistence of the virus in other cell types that support productive infection but at the expense of KP mediated neurotoxicity. KP products can cross an intact blood–brain barrier and then serve as extra substrates for QUIN production by perivascular macrophages and microglia^18^, ^47^ thereby potentially explaining brain injury without brain infection. Moreover, there is some evidence that chronic exposure to QUIN can damage the blood brain barrier allowing ingress of immune related toxins including KP products and QUIN to traffic to the CNS where QUIN^50^ has a neurotoxic effect. KP activation may also have a direct antiviral role through QUIN induced production of type I interferon via N‐methyl‐d‐aspartate receptor activation with Ca^2+^ influx activating Calcium/calmodulin‐dependent protein kinase /interferon regulatory factor 3.^51^

Our study has several limitations. Our sample size has a medium size when considering the longitudinal design (total cognitive and KP datapoints = 349–512 and 59 participants being at least impaired once across the study period), but the cognitive data were missing completely at random (MCAR). The biomarkers had no missing data, while the KP was assessed in all participants 4 times (128 × 4 = 512). Our sample is restricted to the socio‐economically advantaged parts of Sydney and with relatively high education, optimal care access, and management of medical comorbidities, and our study took place prior to the availability of (SARS‐CoV‐2) vaccines. Therefore, generalization of the current findings to less advantaged or vaccinated populations is not possible, although our study provides a unique window into the natural history of COVID‐19. The study has no contemporaneous healthy controls because of the infectious challenges of the pandemic. However, we used Australian pre‐COVID normative data for cognition that was closely comparable to the demographics of the current sample. Furthermore, we used age‐norms data for the KP in addition to control data in healthy controls and other coronaviruses samples. Our study included patients enrolled prior to COVID‐19 vaccination availability; thus, findings may vary among vaccinated populations or with different variants. Despite a carefully conceived PASC definition which associated with disease severity and IFN‐beta, it is likely that the multiple phenotypes and pathophysiology underlying the condition^52^ weakened the association with objective cognitive impairment. As for other infectious condition that impact the brain,^53^ the integration of objective cognitive and olfaction assessments into novel PASC definitions^26^ will be important.

In conclusion, objective cognitive impairment is relatively common post‐acute SARS‐CoV‐2 infection and persists at 12 months and is uniquely associated with KP activation. This lays the foundation for the KP as a potential diagnostic and monitoring as well as a possible therapeutic target. The inclusion of objective testing for cognition and olfaction is needed for improving the accuracy and reliability of PASC as recommended in the International Neuropsychology COVID‐19 taskforce.^26^

## Author Contributions

LC, GG, and BB contributed to the conception and design of the study; LC, DJ, DD, AB, GD, GM, SB, YA BH, SC, MD, ASP, and CP contributed to the acquisition and analysis of data; LC, DJ, DD, AB, GD, GM, SB, YA BH, SC, MD, ASP, CP, GG, AK, and BB contributed to drafting the text or preparing the figures.

## Conflict of Interest

Nothing to report.

## Supporting information


**Table S1.** Acute COVID‐19 illness characteristics by disease severity.
**Table S2**. Cognition and olfaction scores at 2, 4, and 12 months.
**Table S3**. KP metabolites and cytokine original and log‐transformed values, and by reference range cutoffs over time.
**Table S4**. Effect on cognition by biomarker and biomarker * time interaction.
**Table S5**. Effect on KP metabolites (KYN and QUIN) by biomarker and biomarker * time interaction.
**Table S6**. Studies that have objectively assessed cognitive functions post‐acutely in COVID‐19 patients. STROBE statement: Reporting guidelines checklist for cohort, case–control and cross‐sectional studies.

## Data Availability

Data will be made available upon request to the ADAPT study steering committee. The contact is the study PI: Gail Matthews: gmatthews@kirby.unsw.edu.au. Data are in a deidentified format and include a data dictionary. The study protocol and statistical plan for the NeuroCOVID sub‐study as approved by the ADAPT study steering committee will be made available upon request to the ADAPT study PI (Gail Matthews: gmatthews@kirby.unsw.edu.au). The ADAPT study steering committee is in charge of reviewing and approval all research projects associated with the ADAPT study data.
