# Supplementary material for: The kynurenine pathway relates to post‐acute COVID‐19 objective cognitive impairment and PASC
Source: Ann Clin Transl Neurol. 2023 Jun 15;10(8):1338–52. doi: 10.1002/acn3.51825 (PMC10424655; doi:10.1002/acn3.51825)
Supplement: Supplementary file 1 — Table S1. Acute COVID‐19 illness characteristics by disease severity. Table S2. Cognition and olfaction scores at 2, 4, and 12 months. Table S3. KP metabolites and cytokine original and log‐transformed values, and by reference range cutoffs over time. Table S4. Effect on cognition by biomarker and biomarker * time interaction. Table S5. Effect on KP metabolites (KYN and QUIN) by biomarker and biomarker * time interaction. Table S6. Studies that have objectively assessed cognitive functions post‐acutely in COVID‐19 patients. STROBE statement: Reporting guidelines checklist for cohort, case–control and cross‐sectional studies. [file ACN3-10-1338-s001.docx]

**Supplemental Material**

**Additional information on assessments, KP quantifications and statistical plan**

*Olfaction, function, and mental health assessments:* These assessments have been also described in^1^. Olfaction was assessed with The NIH Toolbox Odor Identification Test^2^. Functional status was assessed using the four following statements as part of the ADAPT protocol^1^: I have fully recovered after COVID-19; I feel confident returning to my pre-COVID work; I have returned to my usual activities of daily living; I have returned to my normal exercise level. For each statement, the participant rated whether they strongly agree, agree, slightly agree, slightly disagree, disagree, or strongly disagree, rated from 1 to 6. For analysis we transformed the data into a discrete outcome: agree and disagree. Mental health assessment included three tools to capture anxio-depressive symptoms and post-traumatic disorder (the Depression in the Medically Ill, The Impact of Event Scale Revised and the Anxiety-Depression Subscale of the Somatic and Psychologic Health Report-34). We used a Principal Component analysis to reduce the mental health data to a single component which explained 80% of the mental health data’s variance. Having a pre-existing mental health condition was highly associated with the mental health component (p<.001).

2-month post diagnosis % predicted Total Lung Capacity (TLC) and % predicted Hemoglobin-Corrected Diffusing Capacity of the Lung for Carbon Monoxide (DLCO) extra information and rationale

We selected the two outcomes that were most related to disease severity in the 2021 study analyses: at 2-month post diagnosis % predicted Total Lung Capacity (TLC) and % predicted Haemoglobin-Corrected Diffusing Capacity of the Lung for Carbon Monoxide (DLCO). The study started when the knowledge about COVID-19 infectious potential and associated mortality were still being gathered. Hence, while the study was conducted, several decisions were made to ensure safety of both patients and study personnel and importantly followed the biosafety rules of the Sydney St. Vincent’s Hospital. Therefore, it was impossible to perform full lung function testing during the acute phase because these are aerosol-generating procedures which we weren’t allowed to at this time in Australia in a public hospital. We opted for a 2-month time point to give us the closest estimate of acute disease and the severity of sub-acute pulmonary physiologic abnormalities. Further, we recorded detailed data about the acute infection period. Next, we used population-derived reference equations to generate predicted lung function values for each patient based on their age, sex and height. Then there are several ways to define abnormal lung function, A. Values that are below the lower limit of normal (LLN), B. Standard clinical cut-offs (ie. <80% for TLC and <75% for DLCO are generally accepted) or C. Z-Scores <-1.64. We note that the cut-off of 90% is not supported by the literature and we used the following equations (see^3^ for TLC and see^4^ for DLCO).

PASC definition

PASC was primarily based on the WHO definition (continuation or development of new symptoms 3 months after the initial SARS-CoV-2 infection, with these symptoms lasting for at least 2 months with no other explanation, that is acute and persistent symptoms) based on 18 symptoms by self-report following the post-infectious fatigue research approach^5^ (see legend of figure 1 and supplementary material). We partially modified the WHO definition to include incident late period PASC as defined by^6^ because our study collected self-report at 2-, 4-, 8- and 12 months. The study^6^ which found that incident symptoms should also be considered also focused on the Alpha and Delta variants similar to our study. In addition, at 4-, 8- and 12-months post diagnosis, the participants completed a fatigue scale, and another scale measuring disability for work, everyday activity, and exercise. Self-reported cognitive symptoms were assessed with Patient Assessment of Own’s Functioning Inventory (PAOFI)^7^ at 2-month and at least one more time across the study period for participants with follow-up. The PAOFI is a widely used standard assessment of cognitive symptoms by self-report covering working memory, learning and memory, language, executive functions, sensation (vision), and motor functions (use of hands), A total PAOFI >4 identified at least mildly elevated cognitive symptoms. We further operationalized the WHO definition as in^5^, where it is required that associated symptoms’ disability is assessed. Disability was defined as at least mild impact on work, everyday function, or exercise. Therefore, we classified participants as having PASC (n=70, 55%) or not having PASC (n=57, 45%). We also classified participants as having PASC “only” (n=33, 26%), PASC with disability (n=37, 29%) or not having PASC (n=57, 45%). The current PASC definition was associated with disease severity and highly associated (p<.0001) with the PASC definition used in a subset of the cohort where PASC was associated with chronic immune dysregulation^8^

**Quantification of biomarkers**

Quantification of IFN-β: IFN-β was quantified via LEGENDplex Human Anti-Virus Response Panel as previously described^8^.

Cytokines and chemokines assay: The quantification of the cytokines and chemokines in the serum was carried out by Eve Technologies (Calgary, Canada) using the human focused 15-Plex Discovery Assay. The cytokines and chemokines that were quantified include GMCSF, IFN-γ, Interleukin-1beta (IL-1β), IL-1 receptor antagonist (IL-1 Ra), IL-2, IL-4, IL-5, IL-6, IL-8, IL-10, IL-12p40, IL-12p70, IL-13, MCP-1, and TNF-α.

Enzyme-linked immunosorbent assay: Human GFAP and S100B enzyme-linked immunosorbent assay kits were purchased from R&D Systems (Minneapolis, MN, USA), and NfL enzyme-linked immunosorbent assay kit was purchased from Novus Biologicals (Centennial, CO, USA). The measurement of these proteins in serum was carried out according to manufacturer’s instructions and the concentrations of GFAP, NfL, and S100b were calculated based on the optical density of the samples to the standard curve.

Quantification of the KP metabolites by Ultra High-Performance Liquid Chromatography (uHPLC)

The KP was quantified using Ultra High Performance Liquid Chromatography (uHPLC) as previously described^9^. Approximately 180 µL of patient serum was deproteinized by adding equal volume of with 10% (w/v) trichloroacetic acid. After, the mixture was centrifugated at 4^o^C for 10 min at 12,000 rpm and filtered using 0.20μm polytetrafluoroethylene syringe filters (Millex, Merck) into a new analyzer vial. The KP metabolites were analyzed using an Agilent 1290 series uHPLC system that includes a temperature-controlled column compartment (40^o^ C) and auto-sampler (4^o^ C), diode array (G4212A; Agilent) and fluorescence detector (G1321B; xenon flash lamp; Agilent). The mobile phase was 0.1 M sodium acetate at pH 4.6 and the assay was run with an isocratic flow rate of 0.75ml/min in an ZORBAX Rapid Resolution High Definition C18 column (2.1 mm x 150 mm, 1.8 μm particle size; Agilent) for 12 min. The fluorescence detector was set at excitation/emission wavelength of 280nm/438nm for detection of tryptophan (TRP) and 320 nm/438 nm for detection of 3-hydroxyanthranilic acid (3HAA) and anthranilic acid (AA). Kynurenine (KYN) and 3-hydroxykynurenine (3HK) were detected using a UV detector set to measure absorbance at 365 nm (reference signal off). The results were calculated by interpolation using a six-point calibration curve and expressed as μmol/L or nmol/L. The chromatogram output of KP metabolites was analyzed using the Agilent OpenLAB CDS ChemStation (Edition C.01.04).

Quantification of the KP metabolites by Gas Chromatography/Mass Spectrometry (GC/MS): Agilent 7890 A GC coupled with Agilent 5975 C MS detector and a DB-5MS column (0.25 mM film thickness, 0.25 mm x 30 m capillary column) was used to quantify picolinic acid (PIC) and quinolinic acid (QUIN). Fifty µL of deproteinized serum used in uHPLC was mixed with deuterated internal standards respective to the metabolites of interest were derivatized for quantification. For the sample derivatization the deproteinized samples and deuterated internal standards were dried under vacuum and derivatized with trifluoroacetic anhydride for 10 min at 60^o^C followed by 1,1,1,3,3,3hexafluoroisopropanol for extra 10 min at 60^o^C. Fluorinated esters were then extracted into toluene and washed with 5% sodium bicarbonate. The upper organic layer was collected and washed with 1 mL MilliQ water, and dried using sodium sulphate packed pipette tips. Samples were then transferred into a new analyzer vial for analysis and injected under a splitless mode onto the GC capillary column. The analysis was carried out with the MS operating in negative chemical ionization mode. Selected ions (m/z 273 for PIC, m/z 277 for 4-PIC, m/z 467 for QUIN and m/z 470 for d3-QUIN) were simultaneously monitored. GC oven settings were as follows: oven temperature was held at 75˚C for 3 min and then ramped to 290˚C at a rate of 25˚C/min and held at 290˚C for 4 min for a total run time of 15.6 min. A series of mixed non-deuterated and deuterated standards of PIC and QUIN were used for a six-point standard curve to interpolate the quantity of the sample readout. Levels of PIC and QUIN were calculated and expressed as nmol/L. The chromatogram output of PIC and QUIN was analyzed using Agilent GC/MSD ChemStation software (Edition 02.02.1431).

**Additional information for the statistical analyses**

Data formatting, transformation, and missing data analysis

There were no missing data for cognition or the KP data. IFN-β was measured concomitantly to the 2-month post diagnosis cognitive session in a representative sub-sample of n=62^8^. Other cytokines, inflammatory biomarkers and peripheral biomarkers of brain injury had no missing data and the lowest plausible value was imputed when the product was below detection. One case had pre-morbid anosmia and thus no smell data was collected. Eight cases did not have follow-up olfaction data at 12 months due to technical issues. Attrition in the proportion undergoing neurocognitive assessment (lost to follow-up and/or not yet completed follow-up) was relatively small, (N=1 at 4 month, and N=10 at 12 months) and were completely missing at random (Little's MCAR test, p=10). In addition, cases who remained in the study did not significantly differ in any characteristics compared to those who were not assessed at follow-up.

Prior to analyses, the KP data were inspected for outliers and data were winsorized in 7 instances to minimize the effects of these outliers. To approximate the Normal Distribution, the data was Log transformed for PIC, QUIN, 3HK, AA, and 3HAA. A square root (√) transformation was applied to KYN. Because the KYN showed somewhat binomial distribution which only slightly improved with the square root transformation, we also dummy coded the KYN as 0=within normal range, and 1=KYN >3uM (cut-off based on aged norms^10, 11^). The raw TRP was normally distributed.

Covariates

To reduce the number of comparisons on the main model testing the KP and cognition when also testing a covariate, we conducted a random variable selection of the study covariates (p<.10; linear stepwise regression with forward combine entry and minimum BIC for olfaction, mental health, medical comorbidities, lung function, sex, and disease severity) time effect and cognition over time. We only included mental health at 2-month post diagnosis because at follow-up, mental health symptoms significantly decreased across the sample. PASC definitions were considered a priori, that is not entered in the random selection analyses.

Cognitive definitions for the cross-sectional and longitudinal cognitive analyses

In our study, we used two cognitive outcomes: 1. cognitive impairment: dichotomous definition of impaired cognition compared to normed cut-off at the individual level which detects at least mild cognitive impairment across the Cogstate battery (GDS>.5^12^). We then used this definition i). at 2-month post-infection, ii). as being impaired at least once across the study period (in the context of data MCAR, this definition can be used, and iii). as being impaired (dummy variable) over the study period in a mixed effect regression model. Follow-ups were inclusive of the practice effect correction; 2. Function: practice effect corrected mean z-score that is the continuous performance across the possible range of performance at the group level.

Mixed effect models rationale

For all mixed effect models, we sought to target an optimal balance between parsimony and good fit. Residual maximum likelihood (REML) was used in estimating and reporting all model parameters. Denominator degrees of freedom were estimated using the Satterthwaite method. Graphically, time effect is represented by month-visit, that is 2, 4, 8 (for the KP), and 12 months post diagnosis. Depending on the models’ aim, time is either used as visit time or centered so that the first visit becomes the reference. When relevant, time is also used as a continuous fixed effect and/or random nested effect as the exact month (2 decimals) post diagnosis for each participant. Continuous time was also centered using the sample’s mean. Polynomial time effects (square, quadratic and cubic) were tested and kept in the models when they significantly improve the model’s fit. The significance of new fixed effects was evaluated with univariate and multivariate Wald tests. The significance of the random effect was evaluated using -2LL ratio. Identity and diagonal repeated covariance matrices fitted our data best. For each model, the time fixed effect was tested using a Wald test (set at p<.05 for significance). Random effects were tested using a -2LL ratio (smaller is better). Overall model fit was tested using the AICc (smaller is better).

References

1. Darley DR, Dore GJ, Cysique L, et al. Persistent symptoms up to four months after community and hospital-managed SARS-CoV-2 infection. Med J Aust. 2021 Apr;214(6):279-80.

2. Dalton P, Doty RL, Murphy C, et al. Olfactory assessment using the NIH Toolbox. Neurology. 2013 Mar 12;80(11 Suppl 3):S32-6.

3. Stocks J, Quanjer PH. Reference values for residual volume, functional residual capacity and total lung capacity. ATS Workshop on Lung Volume Measurements. Official Statement of The European Respiratory Society. Eur Respir J. 1995 Mar;8(3):492-506.

4. Stanojevic S, Graham BL, Cooper BG, et al. Official ERS technical standards: Global Lung Function Initiative reference values for the carbon monoxide transfer factor for Caucasians. Eur Respir J. 2017 Sep;50(3).

5. Selvakumar J, Havdal LB, Drevvatne M, et al. Prevalence and Characteristics Associated With Post–COVID-19 Condition Among Nonhospitalized Adolescents and Young Adults. JAMA Network Open. 2023;6(3):e235763-e.

6. Horberg MA, Watson E, Bhatia M, et al. Post-acute sequelae of SARS-CoV-2 with clinical condition definitions and comparison in a matched cohort. Nature Communications. 2022 2022/10/12;13(1):5822.

7. Richardson-Vejlgaard R, Dawes S, Heaton RK, Bell MD. Validity of cognitive complaints in substance-abusing patients and non-clinical controls: the Patient's Assessment of Own Functioning Inventory (PAOFI). Psychiatry Res. 2009 Aug 30;169(1):70-4.

8. Phetsouphanh C, Darley DR, Wilson DB, et al. Immunological dysfunction persists for 8 months following initial mild-to-moderate SARS-CoV-2 infection. Nat Immunol. 2022 Feb;23(2):210-6.

9. Lim CK, Bilgin A, Lovejoy DB, et al. Kynurenine pathway metabolomics predicts and provides mechanistic insight into multiple sclerosis progression. Sci Rep. 2017 Feb 3;7:41473.

10. Heyes MP, Saito K, Lackner A, Wiley CA, Achim CL, Markey SP. Sources of the neurotoxin quinolinic acid in the brain of HIV-1-infected patients and retrovirus-infected macaques. Faseb j. 1998 Jul;12(10):881-96.

11. Badawy AAB, Guillemin G. The Plasma [Kynurenine]/[Tryptophan] Ratio and Indoleamine 2,3-Dioxygenase: Time for Appraisal. Int J Tryptophan Res. 2019;12:1178646919868978-.

12. Kamminga J, Bloch M, Vincent T, Carberry A, Brew BJ, Cysique LA. Determining optimal impairment rating methodology for a new HIV-associated neurocognitive disorder screening procedure. J Clin Exp Neuropsychol. 2017 Oct;39(8):753-67.

**Table S1: Acute COVID-19 Illness Characteristics by Disease Severity**

| Acute Illness Characteristics | Full Sample | | Mild | | Moderate | | Severe | |
| --- | --- | --- | --- | --- | --- | --- | --- | --- |
|  | *n* | *%* | *n* | *%* | *n* | *%* | *n* | *%* |
| Acute Symptom Severity | 127 | 100.0 | 49 | 38.6 | 66 | 52.0 | 12 | 9.4 |
| **Fever/Chills** | 61 | 48.0 | 9 | 18.4 | 45 | 68.2 | 7 | 58.3 |
| **Cough** | 80 | 63.0 | 18 | 36.7 | 54 | 81.8 | 8 | 66.7 |
| **Haemoptysis** | 1 | 0.8 | 0 | 0.0 | 1 | 1.5 | 0 | 0.0 |
| **Runny Nose** | 45 | 35.4 | 12 | 24.5 | 29 | 43.9 | 4 | 33.3 |
| **Anosmia** | 54 | 42.5 | 16 | 32.7 | 35 | 53.0 | 3 | 25.0 |
| **Ageusia** | 56 | 44.1 | 17 | 34.7 | 35 | 53.0 | 4 | 33.3 |
| **Sore Throat** | 53 | 41.7 | 14 | 28.6 | 32 | 48.5 | 7 | 58.3 |
| **Shortness of Breath** | 48 | 37.8 | 4 | 8.2 | 38 | 57.6 | 6 | 50.0 |
| **Chest Pain** | 23 | 18.1 | 3 | 6.1 | 16 | 24.2 | 4 | 33.3 |
| **Nausea/Vomiting** | 25 | 19.7 | 1 | 2.0 | 19 | 28.8 | 5 | 41.7 |
| **Diarrhoea** | 32 | 25.2 | 1 | 2.0 | 25 | 37.9 | 6 | 50.0 |
| **Headache** | 82 | 64.6 | 26 | 53.1 | 49 | 74.2 | 7 | 58.3 |
| **Muscle Aches** | 68 | 53.5 | 19 | 38.8 | 43 | 65.2 | 6 | 50.0 |
| **Joint Aches** | 48 | 37.8 | 14 | 28.6 | 29 | 43.9 | 5 | 41.7 |
| **Weakness** | 65 | 51.2 | 17 | 34.7 | 44 | 66.7 | 4 | 33.3 |
| **Fatigue** | 98 | 77.2 | 33 | 67.3 | 55 | 83.3 | 10 | 83.3 |
| **Altered Consciousness/Confusion** | 12 | 9.4 | 0 | 0.0 | 9 | 13.6 | 3 | 25.0 |
| **Other** | 38 | 29.9 | 16 | 32.7 | 20 | 30.3 | 2 | 16.7 |
| Admitted to Intensive Care Unit | 4 | 3.1 | 0 | 0 | 0 | 0 | 4 | 3.1 |
| Acute Respiratory Distress Syndrome (ARDS) | 3 | 2.3 | 0 | 0 | 0 | 0 | 3 | 2.3 |
| **Bolded** symptoms were also collected at 2-, 4-, 8- and 12-months to determine PASC in addition to the fatigue, disability, and cognitive symptoms scales | | | | | | | | |

**Table S2: Cognition and olfaction scores at 2-, 4- and 12-months**

| **Test/measure** | **2 Months** | | **4 Months** | | **12 Months** | |
| --- | --- | --- | --- | --- | --- | --- |
|  | ***n*** | ***M (SD)*** | ***n*** | ***M (SD)*** | ***n*** | ***M (SD)*** |
| **Cognition (CogState Computerized Battery)** |  |  |  |  |  |  |
| **Detection Speed z-score** | 127 | -0.54 (0.70) | 121 | -0.69 (0.65) | 101 | -0.88 (0.76) |
| **Identification Speed z-score** | 127 | 0.19 (0.59) | 121 | 0.07 (0.64) | 101 | -0.06 (0.62) |
| **One Back Speed z-score** | 127 | -0.12 (1.13) | 121 | -0.33 (1.01) | 101 | -0.39 (0.98) |
| **One Card Learning Accuracy z-score** | 127 | 0.63 (1.09) | 121 | 0.09 (1.13) | 101 | 0.10 (1.30) |
| **One Back Accuracy z-score** | 127 | -0.04 (0.94) | 121 | -0.05 (0.92) | 101 | -0.10 (1.00) |
| **Global Mean z-score** | 127 | 0.02 (0.50) | 121 | -0.18 (0.52) | 101 | -0.26 (0.55) |
| **Olfaction NIH Odor Identification Test** |  |  |  |  |  |  |
| **Global Mean T-score** | 126 | 44.20 (10.25) | 119 | 46.05 (9.43) | 93 | 44.00 (9.50) |

Cognition data have been corrected for task-specific practice effects and demographically adjusted for age, sex, and education. Olfaction data is corrected for age, sex, education, and race/ethnicity (i.e., white/other). Two cases had premorbid anosmia and were not tested. At 12 months, 8 cases missed their olfaction assessment due to technical issues.

**Table S3: KP metabolites and cytokine original and log-transformed values, and by reference range cut-offs over time**

|  | | **2-month post diagnosis** | | | | **4-month post diagnosis** | | | |  |  |
| --- | --- | --- | --- | --- | --- | --- | --- | --- | --- | --- | --- |
| **Biomarker** | | **Original** | **Transformed** | **% Elevated^1^** | **Original** | | **Transformed** | **% Elevated^1^** | **Lower limit of detection on original value set at pg/mL (detection rate max – min or mean over study period)** | | |
| n | |  | 126 |  |  | | 122 |  |  | | |
|  |  |  | M (SD) |  |  | | M (SD) |  |  | | |
|  | NFL | 9.57 (16.22) | 1.95 (0.74) | 1.5% | 10..17 (17.40) | | 1.96 (0.77) | 3.2% | 8 (not detected: 80% – 54%) | | |
|  | GFAP | 1232.25 (3593) | 6.30 (1.77) | 31% | 917.06 (1977.38) | | 6.21 (1.67) | 30% | 30 (not detected: 44% - 37%) | | |
|  | S100B | 27.58 (47.98) | 3.02 (0.57) | 3.2% | 35.16 (73.85) | | 3.05 (0.78) | 5.7% | 20 (not detected: 65% - 20%) | | |
|  | GMCSF | 12.91 (34.22) | -0.79 (3.44) | - | 39.21 (154.76) | | -0.13 (3.58) | - | 0.01 (not detected: 35%) | | |
|  | FNγ | 2.14 (5.21) | -0.10 (1.20) | - | 2.88 (8.51) | | -0.13 (1.34) | - | (Detected in 100%) | | |
|  | IL1β | 58.68 (103.21) | 3.16 (1.31) | - | 68.87 (157.67) | | 3.08 (1.43) | - | (Detected in 99.9%) | | |
|  | IL1Ra | 14.04 (23.51) | 2.03 (0.98) | - | 14.66 (36.36) | | 1.79 (1.10) | - | (Detected in 100%) | | |
|  | IL2 | 3.15 (8.48) | -0.75 (1.86) | - | 4.43 (13.87) | | -0.88 (2.07) | - | (Detected> 80%) | | |
|  | IL4 | 0.80 (1.70) | -2.43 (2.08) | - | 0.96 (2.25) | | -2.39 (2.17) | - | 0.02 (not detected 60%) | | |
|  | IL5 | 4.38 (8.44) | 1.05 (0.79) | - | 3.83 (7.43) | | 0.86 (0.86) | - | (Detected: 100%) | | |
|  | IL6 | 2.39 (12.70) | -0.42 (1.22) | - | 2.99 (16.81) | | -0.48 (1.31) | - | (Detected: 100%) | | |
|  | IL8 | 9.58 (8.13) | 2.00 (0.72) | - | 8.38 (10.45) | | 1.82 (0.75) | - | (Detected: 100%) | | |
|  | IL10 | 2.26 (6.95) | -1.32 (2.30) | - | 1.58 (4.23) | | -1.81 (2.26) | - | 0.01 (not detected: 40%) | | |
|  | L12p40 | 115.13 (125.03) | 4.44 (0.77) | - | 100.77 (146.46) | | 4.25 (0.83) | - | (Detected: 100%) | | |
|  | IL12p70 | 6.55 (23.89) | -0.28 (2.00) | - | 7.89 (25.61) | | -0.34 (2.09) | - | 0.1 (not detected: 30%) | | |
|  | IL13 | 48.67 (72.79 | 2.40 (2.02) | - | 54.44 (82.82) | | 2.10 (2.26) | - | (Detected: >80%) | | |
|  | TNFα | 39.31 (44.45) | 3.39 (0.71) | - | 36.56 (49.48) | | 3.25 (0.74) | - | (Detected: 100%) | | |
|  | MCP-1 | 268.50 (102.56) | 268.50 (102.56) | - | 242.46 (87.65) | | 242.46 (87.66) | - | (Detected: 99.9%) | | |
|  | IFN-β^2^ | 117.17 (319.93) | 3.84 (1.15) | - | - | | - | - | (Detected: 100%) | | |

^1^ Reference range cut-offs

^2^ n = 62, only measured at time 1.

|  | | **8-month post diagnosis** | | | **12-month post diagnosis** | | |
| --- | --- | --- | --- | --- | --- | --- | --- |
| **Biomarker** | | **Original** | **Transformed** | **% Elevated^1^** | **Original** | **Transformed** | **% Elevated^1^** |
| n | |  | 114 |  |  | 84 |  |
|  |  |  | M (SD) |  |  | M (SD) |  |
|  | NFL | 14.59 (23.50) | 2.27 (0.73) | 7% | 45.03 (106.98) | 2.82 (1.20) | 27% |
|  | GFAP | 1317 (3479.06) | 6.36 (1.86) | 33% | 1275.58 (3194) | 6.15 (2.04) | 36% |
|  | S100B | 52.78 (169.11) | 3.17 (0.84) | 7% | 63.55 (146.63) | 3.13 (1.46) | 12% |
|  | GMCSF | 27.55 (117.40) | 0.03 (3.50) | - | 22.76 (68.59) | -0.54 (3.70) | - |
|  | IFNγ | 1.71 (2.45) | -0.15 (1.14) | - | 2.08 (2.94) | 0.03 (1.16) | - |
|  | IL1β | 62.12 (118.76) | 3.17 (1.37) | - | 66.39 (135.41) | 3.32 (1.25) | - |
|  | IL1Ra | 12.48 (24.07) | 1.82 (1.03) | - | 14.22 (33.36) | 2.01 (0.92) | - |
|  | IL2 | 3.57 (10.46) | -0.60 (1.90) | - | 4.32 (13.56) | -0.41 (1.88) | - |
|  | IL4 | 0.61 (1.36) | -2.57 (1.97) | - | 0.78 (1.53) | -2.46 (2.14) | - |
|  | IL5 | 3.56 (4.57) | 0.94 (0.75) | - | 4.16 (6.22) | 0.97 (0.88) | - |
|  | IL6 | 2.60 (16.68) | -0.46 (1.19) | - | 1.089 (1.33) | -0.42 (1.00) | - |
|  | IL8 | 8.58 (9.27) | 1.85 (0.79) | - | 8.62 (10.77) | 1.83 (0.79) | - |
|  | IL10 | 1.79 (4.53) | -1.70 (2.31) | - | 2.49 (5.93) | -1.32 (2.36) | - |
|  | IL12p40 | 105.14 (152.22) | 4.23 (0.81) | - | 139.11 (195.72) | 4.48 (0.85) | - |
|  | L12p70 | 4.09 (10.87) | -0.29 (1.83) | - | 4.99 (14.25) | -0.17 (1.89) | - |
|  | IL13 | 38.15 (35.65) | 2.16 (2.10) | - | 46.85 (50.81) | 2.26 (2.16) | - |
|  | TNFα | 32.07 (22.09) | 3.29 (0.62) | - | 37.11 (31.15) | 3.41 (0.60) | - |
|  | MCP-1 | 254.33 (93.76) | 254.33 (93.76) | - | 246.13 (89.82) | 246.13 (89.82) | - |

^1^ Reference range cut-offs (NFL ≥ 35 pg/mL; GFAP ≥ 450 pg/mL; S100B > 100 pg/mL

**Table S4: Effect on Cognition by biomarker and biomarker * time interaction**

| Biomarker | Random intercept | Main time effect | Main Biomarker effect | Interaction | -2LL | AICc |
| --- | --- | --- | --- | --- | --- | --- |
|  | B (SE) p | B (SE) p | B (SE) p | B (SE) p |  |  |
| Log NFL | .173 (.028) <.001 | -.034 (.013) .007 | -.008 (.029) .773 | .005 (.005) .355 | 450 | 460 |
| Log GFAP | .14 (.029) <.001 | -.021 (.019) .283 | -.020 (.022) .362 | -.001 (.003) .766 | 221 | 231 |
| Log S100B | .174 (.028) <.001 | -.025 (.016) .112 | .022 (.032) .485 | .001 (.005) .920 | 450 | 460 |
| Log GMCSF | .172 (.028) <.001 | -.023 (.004) <.001 | -.020 (.009) .*024* | -.001 (.001) .592 | 410 | 420 |
| Log IFNγ | .175 (.028) <.001 | -.023 (.004) <.001 | -.033 (.026) .198 | -.006 (.003) .112 | 407 | 417 |
| Log IL1β | .172 (.028) <.001 | -.013 (.012) .271 | -.015 (.026) .574 | -.003 (.003) .366 | 410 | 420 |
| Log IL1Ra | .172 (.028) <.001 | -.017 (.010) .080 | .018 (.032) .583 | -.003 (.005) .515 | 409 | 419 |
| Log IL2 | .173 (.028) <.001 | -.024 (.004) <.001 | -.015 (.017) .373 | -.003 (.002) .244 | 411 | 421 |
| Log IL4 | .173 (.028) <.001 | -.024 (.006) <.001 | .001 (.017) .938 | <.001 (.002) .943 | 413 | 423 |
| Log IL5 | .171 (.028) <.001 | -.024 (.006) <.001 | -.011 (.037) .775 | .001 (.005) .806 | 409 | 419 |
| Log IL6 | .175 (.028) <.001 | -.025 (.004) <.001 | -.043 (.028) .132 | -.005 (.004) .221 | 408 | 418 |
| Log IL8 | .172 (.028) <.001 | -.019 (.011) .095 | .015 (.045) .732 | -.002 (.006) .713 | 409 | 419 |
| Log IL10 | .172 (.028) <.001 | -.028 (.005) <.001 | <.001 (.015) .997 | -.003 (.002) .055 | 410 | 420 |
| Log IL12p40 | .17 (.028) <.001 | -.018 (.023) .417 | .071 (.042) .088 | -.001 (.005) .815 | 407 | 417 |
| Log IL12p70 | .171 (.028) <.001 | -.023 (.004) <.001 | -.027 (.016) .103 | <.001 (.002) .943 | 410 | 420 |
| Log IL13 | .172 (.028) <.001 | -.020 (.006) .001 | -.003 (.015) .859 | -.001 (.002) .505 | 413 | 423 |
| Log TNFα | .173 (.028) <.001 | -.017 (.022) .458 | -.005 (.049) .923 | -.002 (.007) .770 | 409 | 419 |
| MCP-1 | .173 (.028) <.001 | -.026 (.012) .028 | .001 (<.001) .047 | <.001 (<.001) .679 | 425 | 435 |

FDR across all fixed effects (time, main cytokine, and interaction): *p*<0.001

**Table S5: Effect on KP metabolites (KYN and QUIN) by biomarker and biomarker * time interaction**

| **KP metabolite** | **Biomarker** | **Random intercept** | **Main time effect** | **Main biomarker effect** | **Interaction** | **-2LL** | **AICc** |
| --- | --- | --- | --- | --- | --- | --- | --- |
|  |  | **B (SE) p** | **B (SE) p** | **B (SE) p** | **B (SE) p** |  |  |
| **√KYN** | Log NFL | - | -.077 (.018) <.001 | .032 (.044) .474 | -.004 (.008) .584 | 900 | 910 |
|  | Log GFAP | - | -.113 (.03) <.001 | .015 (.025) .546 | .003 (.005) .47 | 529 | 539 |
|  | LogS100B | - | -.068 (.025) .006 | .007 (.043) .878 | -.006 (.008) .449 | 899 | 909 |
|  | Log GMCSF | - | -.085 (.007) <.001 | .031 (.009) .001 | -.003 (.002) .107 | 892 | 902 |
|  | Log IFNγ | - | -.086 (.007) <.001 | .008 (.026) .754 | .007 (.005) .171 | 896 | 906 |
|  | Log IL1β | - | -.109 (.018) <.001 | .008 (.024) .741 | .007 (.005) .173 | 897 | 907 |
|  | Log IL1Ra | - | -.117 (.015) <.001 | -.024 (.032) .461 | .016 (.007) .021 | 893 | 903 |
|  | Log IL2 | - | -.085 (.007) <.001 | .009 (.017) .608 | .001 (.004) .733 | 901 | 911 |
|  | Log IL4 | - | -.081 (.01) <.001 | -.001 (.016) .963 | .002 (.003) .569 | 902 | 912 |
|  | Log IL5 | - | -.101 (.01) <.001 | -.031 (.039) .427 | .016 (.008) .049 | 894 | 904 |
|  | Log IL6 | - | -.086 (.007) <.001 | .070 (.027) .009 | -.003 (.006) .568 | 892 | 902 |
|  | Log IL8 | - | -.103 (.018) <.001 | -.037 (.043) .391 | .009 (.009) .321 | 897 | 907 |
|  | Log IL10 | - | -.081 (.008) <.001 | .009 (.014) .540 | .004 (.003) .130 | 896 | 906 |
|  | Log IL12p40 | - | -.172 (.036) <.001 | -.031 (.04) .438 | .020 (.008) .016 | 892 | 902 |
|  | LognIL12p70 | - | -.085 (.007) <.001 | .003 (.016) .857 | .003 (.003) .343 | 900 | 910 |
|  | Log IL13 | - | -.097 (.01) <.001 | .002 (.015) .908 | .005 (.003) .138 | 898 | 908 |
|  | Log TNFα | - | -.141 (.031) <.001 | -.008 (.045) .864 | .016 (.009) .074 | 894 | 904 |
|  | MCP-1 | - | -.126 (.018) <.001 | -.001 (<.001) .051 | <.001 (<.001) .018 | 912 | 922 |
| **Log QUIN** | Log NFL | .044 (.011) <.001 | -.009 (.001) <.001 | .024 (.026) .346 | -.01 (.005) .066 | 534 | 559 |
|  | Log GFAP | .027 (.013) .042 | -.01 (.002) <.001 | .008 (.016) .63 | .002 (.003) .536 | 334 | 356 |
|  | Log S100B | .039 (.011) <.001 | -.009 (.001) <.001 | -.015 (.026) .575 | -.007 (.005) .163 | 541 | 567 |
|  | Log GMCSF | .042 (.011) <.001 | -.009 (.001) <.001 | .012 (.007) .067 | <.001 (.001) .961 | 548 | 573 |
|  | Log IFNγ | .037 (.011) .001 | -.01 (.001) <.001 | .039 (.019) .043 | .002 (.004) .644 | 542 | 568 |
|  | Log IL1β | .04 (.011) <.001 | -.009 (.001) <.001 | .007 (.018) .709 | -.004 (.004) .318 | 546 | 571 |
|  | Log IL1Ra | .038 (.011) <.001 | -.009 (.001) <.001 | .026 (.023) .266 | -.006 (.005) .215 | 543 | 568 |
|  | Log IL2 | .039 (.011) <.001 | -.009 (.001) <.001 | .014 (.012) .26 | -.003 (.003) .283 | 546 | 571 |
|  | Log IL4 | .039 (.011) <.001 | -.009 (.001) <.001 | -.006 (.012) .631 | -.001 (.002) .514 | 548 | 573 |
|  | Log IL5 | .039 (.011) <.001 | -.009 (.001) <.001 | .028 (.028) .317 | -.002 (.006) .734 | 544 | 569 |
|  | Log IL6 | .033 (.01) .001 | -.01 (.001) <.001 | .079 (.019) <.001 | -.008 (.004) .064 | 525 | 550 |
|  | Log IL8 | .037 (.011) .001 | -.009 (.001) <.001 | .048 (.031) .127 | <.001 (.006) .98 | 542 | 568 |
|  | Log IL10 | .039 (.011) <.001 | -.01 (.001) <.001 | .017 (.01) .101 | .002 (.002) .36 | 545 | 571 |
|  | Log IL12p40 | .038 (.011) <.001 | -.009 (.001) <.001 | .016 (.029) .582 | .008 (.006) .173 | 543 | 568 |
|  | Log IL12p70 | .039 (.011) <.001 | -.009 (.001) <.001 | -.001 (.012) .918 | <.001 (.002) .952 | 548 | 574 |
|  | Log IL13 | .039 (.011) <.001 | -.009 (.001) <.001 | .004 (.011) .724 | .002 (.002) .448 | 548 | 573 |
|  | Log TNFα | .038 (.011) <.001 | -.01 (.001) <.001 | .071 (.035) .043 | .01 (.007) .146 | 539 | 564 |
|  | MCP-1 | .04 (.011) <.001 | -.009 (.001) <.001 | <.001 (<.001) .284 | <.001 (<.001) .019 | 557 | 582 |

FDR across all fixed effects (time, main cytokine, and interaction): *p*<0.001

**Table S6: Studies that have objectively assessed cognitive functions post-acutely in COVID-19 patients.**

|  | | | | | |
| --- | --- | --- | --- | --- | --- |
| **Study** | **Sample** | **Design** | **Timeline** | **Measures** | **Main Findings** |
| **Alemanno et al., 2021**  Italy | 87 patients admitted to the COVID-19 Rehabilitation  4 groups according to the respiratory assistance in the acute phase: Group1 (orotracheal intubation), Group2 (non-invasive ventilation using Biphasic Positive Airway Pressure), Group3 (Venturi Masks), Group4 (no oxygen therapy)  62 Male, mean age 67.23 ± 12.89 years | Longitudinal | Follow-ups were performed at one month after home-discharge  56 patients (22 of Group 1, 12 of Group 2, 20 of Group 3 and 2 of Group 4) | MMSE, MoCA, Hamilton Rating Scale for Depression, and Functional Independence Measure (FIM) | MoCA scores showed that 74.2% Group 1, 94.4% Group 2, 89.6% Group 3 and 77.8% presented with deficits  MMSE 12.9% Group 1 had mild to severe deficits; 55.6% Group 2 had mild to moderate deficits; 48.3% Group 3 had mild to severe deficits; and 44.4% had moderate deficits.  Results were correlated with age  At follow-up overall improvement on MoCA and MMSE, but many deficits remained. Group 1 is the least impaired. |
| **Almeria et al. (2020)**  Spain | 35 hospitalised patients, 54% female. 20% in ICU, 60% oxygen treatment, 20% neither ICU or oxygen treatment. Mean age 47.6 years (SD = 8.9 years)  exclusion of subjects older than 60 or with psychiatric condition | Cross-sectional | 10–35 days post hospital discharge | Visual Reproduction (Wechsler Memory Scale – IV), Verbal Learning via list learning, interference, and recognition | Patients requiring oxygen therapy during hospitalization: impairment in attention, working memory, processing speed, executive function, and global cognition; ICU treated patients: impaired executive dysfunction |
| **Amalakanti et al. (2021)**  India | 93 asymptomatic COVID-19 patients, 52.3% female, mean age 36.2 years (SD = 11.7 years). 102 healthy controls, 54.7% female, mean age 35.6 (SD = 9.8 years). All patients had less than four years of education | Cross-sectional | Unclear if measured post-acutely as details not provided | MoCA | Differences in overall MoCA scores between groups was negligible, although COVID-19 performed significantly lower in visuoperception, naming and fluency domains than that of controls. |
| **Beaud et al. (2021)** Switzerland | 13 previous ICU patients in in-patient rehabilitation, 23% female. Mean age 64.8 years (SD = 7.6 years)  patients still in hospital. Exclusion of patients with pre-existing psychiatric diseases, not controlling for age in FAB | Cross-sectional | Post-critical acute stage of severe COVID-19 (still in hospital) | MoCA, Frontal Assessment Battery (FAB) | Cognitive impairment in 69% (31% mild, 39% moderate to severe). More extensive impairment in executive, memory, attentional and visuospatial functions. 92% impaired in lexical fluency (FAB subtest) |
| **Becker et al. (2021)**  United States | 740 patients, 51% outpatients, 22% emergency department, 27% hospitalised. 63% female, Mean age 49 years (SD = 14.2 years), education: 14% ≤12 years education, 86% > 12 years education | Cross-sectional | 7.6 months (SD = 2.7) after SARS-CoV-2 diagnosis | Number Span Forward and backward, Trail Making Test Part A and B, Phonemic and Category Fluency, Hopkins Verbal Learning Test-Revised.  Did not account for mental health | Deficits across domains: processing speed (18%), executive functioning (16%), phonemic fluency (15%), category fluency (20%), memory encoding (24%), and memory recall (23%). Compared to outpatients, hospitalised patients were more likely to have attention, executive function, category fluency and memory impairments |
| **Bungenberg et al. 2021**  Germany | 50 patients with persisting symptoms for at least 4 weeks were included and classified by initial hospitalization status: hospitalized (*n* = 21) and non‐hospitalized (*n* = 29). Median Age 50.5 years (range 22–84 years), 56% female; median education: 15.5 (12.75–18) | Cross-sectional | Median time from SARS-CoV-2 detection to investigation was 29.3 weeks (range 3.3-57.9) | MoCA, version 7  Test of Attentional Performance (TAP), TMT A and B, Digit span forwards and backwards. Verbal fluencies, Stroop test variant (Farbe‐Wort‐Interferenztest, FWIT), Auditory Verbal Memory Test (VLMT), Rey Complex Figure copy and recall, Boston Naming Test | Mild deficits were found in attention, executive functions, and memory  Hospitalized patients performed worse in global cognition, logical reasoning, and processes of verbal memory. In both groups, fatigue severity was associated with reduced performance in attention and psychomotor speed tasks and reduced quality of life and with more persisting symptoms. |
| **De Lorenzo et al. (2020)**  Italy | 185 patients, 34% female. 68% hospitalised, 32% discharged from emergency department. Mean age 57 years (SD = 10.4 years)  Only patients with suspected pneumonia | Cross-sectional | 3.3 weeks post-discharge from hospital or emergency department | MoCA  Did not account for mental health | Cognitive impairment in 25% |
| **Del Brutto et al. (2021 & 2022)**  Ecuador | 2021: 52 non-hospitalised patients, 63% female. Mean age 62.6 years (SD=11 years). 41 healthy, demographically matched controls.  Including non-hospitalised people aged 40+ years  2022: 78 participants, 50 with history of mild COVID-19 and 28 without | Longitudinal  Longitudinal | First assessment: 2013-2015, Second Assessment: 2017-2019, Third Assessment: 6 months post-infection  2 cognitive testing before the pandemic and 2, 6 and 18 months after the initial SARS-CoV-2 outbreak | MoCA – Spanish Version (Adjusted for demographics, cardiovascular risk factors, mental health, and education)  Same | COVID-19 patients 18 times more likely to develop cognitive decline compared to healthy controls. 12% of patients declined cognitively after infection compared to 2% of healthy controls assessed on same timeline.  Significant-likely age-related-decline in MoCA scores between the two prepandemic tests which did not differ between groups; at 6 months, only COVID-19 survivors showed significant decline, but reversed at 18-month |
| **Frontera et al. (2022)**  USA | N=242 patients (median age 65, 64% male, 34% intubated during hospitalization) and N=174 completed both 6- and 12-month follow-up | Longitudinal | 6- to 12-months post infection | Modified Rankin Score, T-MoCA, Neuro-QoL  Results adjusted for age, sex, race, pre-COVID Modified Ranking Score and intubation status | Those with neurological complications (N=113) had higher fatigue scores but other differences. Significant improvements in outcome trajectories from 6- to 12-months were observed in T-MoCA scores (56% improved, median difference 1 point. and Neuro-QoL anxiety scores (45% improved). Non-significant improvements occurred in fatigue, sleep and depression. |
| **Hampshire et al (2021)** UK | N= 81,337 | Cross-sectional | January and December 2020 | Clinically validated web-optimized assessment (gbit.cognitron.co.uk), and questionnaire items capturing self-report of suspected and confirmed COVID-19 infection and respiratory symptoms | COVID+ showed significant cognitive deficits versus controls when controlling for age, gender, education level, income, racial-ethnic group, pre-existing medical disorders, tiredness, depression and anxiety, and pre-morbid level.  Hospitalized worse than non-hospitalized and controls; non-hospitalized worse than controls |
| **Hellgren et al. (2021).**  Sweden | 35 hospitalised patients, 20% female. Median age 59 years (IQR: 51-66), 19 needed mechanical ventilation, 14 did not require ventilation.  Only including those with concerning results on neuropsychological testing or suspected cognitive impairment | Cross-sectional | Neurocognitive testing: 5 months post-discharge (median 142 days); MRI: 7 months post-discharge (median 217 days). | Repeatable battery for the assessment of neuropsychological status (RBANS) | Cognitive impairment in 46%, of which 29% had severe impairment. Patients with abnormalities on MRI had a significantly (p = .031) lower visuospatial index compared to those with a normal MRI. |
| **Jaywant et al. (2021)**  United States | 57 previous ICU patients in in-patient rehabilitation, 25% female. Mean age 64.5 years (SD = 13.9 years)  Only including patient with suspected cognitive impairment | Cross-sectional | 2 months post hospital admission, still in in-patient rehabilitation | Age-normed Brief Memory and Executive Test (BMET) | Cognitive impairment in 81%, ranging from mild to severe. Common deficits in working memory (55%), set-shifting (47%), divided attention (46%), and processing speed (40%) |
| **Liu et al., 2022**  China | 1438 COVID-19 survivors and 438 control individuals; *all aged 60+*; COVID-19 was categorized as severe or nonsevere following the American Thoracic Society guidelines | Longitudinal | Follow-up at 6 and 12 months | Informant Questionnaire on Cognitive Decline in the Elderly and the Telephone Interview of Cognitive Status-40 | The incidence of cognitive impairment in survivors 12 months after discharge was 12.45%. Individuals with severe cases had lower Telephone Interview of Cognitive Status-40 scores than those with nonsevere cases and control individuals at 12 months. Severe COVID-19 was associated with a higher risk of early-onset cognitive decline, late-onset cognitive decline and progressive cognitive decline while nonsevere COVID-19 was associated with a higher risk of early-onset cognitive decline (adjusted for age, sex, education level, body mass index, and comorbidities) |
| **Mattioli et al. (2021)**  Italy | 120 health care workers who had COVID-19 of mostly mild to moderate severity (2 patients required hospitalisation), 75% female, mean age 47.9 years, mean education 16 years. 30 (controls) health care workers who had not had COVID-19, 73.3% female, mean age 45.7 years, mean education 18 years. | Cross-sectional | Mean of 126 days from diagnosis (range of 12 to 215 days) | Controlled Oral Word Association (COWA), Rey figure copy and recall, California Verbal Learning Test (CVLT), TEA attention test, Tower of London test, MMSE, Depression anxiety and stress scale-21 (DASS-21) | Frequency of neurological deficits and cognitive impairment was negligible. COVID-19 patients were impaired on 1.6 tests on average, and this was not significantly different to those who did not have COVID-19. Anxiety, stress and depression was significantly more elevated in those with COVID-19 than those without COVID-19. |
| **Mattioli et al. (2022)**  Italy | 52 COVID+ cases treated in intensive care unit (ICU patients), 163 not hospitalized (non-ICU patients). | Cross-sectional | 4 months after the diagnosis | Neurological exam and extensive cognitive evaluation, investigating general cognition, memory, verbal fluency, visuospatial abilities and executive functions | eripheral nervous system deficits in 2/163 (1.2%) of non-ICU and in 7/52 (13.5%) of the ICU cases; ICU patients performed significantly worse than non-ICU cases; unrelated to tested comorbid effects |
| **Mazza et al. (2021)**  Italy | 226 patients who initially presented to emergency department, 177 hospitalised, 49 discharged and treated at home. 34% female, mean age 58.5 years (SD = 12.8 years). Only subsample of 130 patients completed BACS.  Exclusion of patients older than 70 | Longitudinal | 1 and 3 months after hospital discharge | Brief Assessment of Cognition in Schizophrenia (BACS) – age normed  Demographics not provided for sample who completed BACS. | 78% of sample had poor performances in at least one cognitive domain, with executive functions and psychomotor coordination impaired in 50% and 57% of the sample respectively at 3 month follow up. Self-reported psychopathology influenced cognition. |
| **Miskowiak et al. (2021)**  Denmark | 29 hospitalised patients, 41% female. 100 healthy age- and education-matched controls | Longitudinal (baseline results) | 3-4 months post hospital discharge | Screen for Cognitive Impairment in Psychiatry - Danish Version (SCIP-D), and the Trail Making Test-Part B.  Did not control for mental health | Cognitive impairment in 59%- 65% (depending on which cutoff was applied). Large effect size for verbal learning and executive functioning. Moderate impairment for working memory, verbal fluency and psychomotor speed |
| **Raman et al. (2021)**  UK | 58 hospitalised patients, 41% female. 95% mechanical ventilation, 36% ICU. Mean age 55.4 years (SD = 13.2 years). 30 uninfected controls, matched for age, sex, BMI and comorbidities | Longitudinal (baseline results) | 2.3 months from disease-onset, (1.6 months post hospital discharge) | MoCA | Impairments in executive and visuospatial domains significantly more common in patients than controls (40% patients vs 16% controls), median MoCA scores in patients not significantly different from controls |
| **Soldati et al. (2021)**  Brazil | 23 ICU patients, 22% female. Median age 53.6 years (IQR ± 11.7)  Only ICU patients with mechanical ventilation | Cross-sectional | 3.3 months post hospital discharge | Telephone Interview of Cognitive Status (TICS)  Did not control for mental health and education | 13% met criteria for mild cognitive impairment, 61% of patients fell within normal limits on cognitive assessments, 26% had ambiguous result. No participants had severe cognitive dysfunction |
| **Tomasoni et al. (2021)**  Italy | 25 hospitalised patients with virological clearance  Time of hospitalisation, disease severity and demographic information not specified | Cross-sectional | 1 – 3 months after virological clearance | MMSE (adjusted for age and education years)  Did not control for mental health | Cognitive impairment in 40%, ranging from mild to severe. 16% had ambiguous results |
| **Woo et al. (2020)**  Germany | 18 patients, 58% female. 61% hospitalised, 39% non-hospitalised, no ICU. Mean age 42.2 years (SD=14.3 years). 10 age-matched control. Controls not matched for sex | Cross-sectional | 2.8 months post recovery | Modified Telephone Interview of Cognitive Status (TICS-M)  Did not account for control for education. | Patients scored significantly lower on TICS-M compared to healthy controls, especially regarding short-term memory, attention, concentration and language. Neuropsychologic deficits were independent from hospitalization and disease severity. |
| **Zhou et al. (2020)**  China | 29 patients, 38% female. Median age 47 years (SD = 10.54 years). 29 age-, sex- and education-matched controls.  Disease severity not reported  Only patients with >9 years education.  Excluded patient with a mental health disorder and left handedness | Cross-sectional | 2-3 weeks post infection | iPad-based online neuropsychological tests, including the Trail Making Test (TMT), Sign Coding Test (SCT), Continuous Performance Test (CPT), and Digital Span Test (DST). | Mild cognitive impairments among patients, mainly affecting sustained attention. No significant difference between patient and healthy controls in TMT, SCT, or DST |

*Note.* This tables features studies assessing adult patients with confirmed SARS-CoV-2 infection (according to World Health Organization (2021a) criteria), using objective cognitive assessments, **censored 31^st^ of March 2022**. Methodology of this literature review included searching on PubMed Database and Google Scholar using the following keywords: neurocognitive functioning COVID-19, cognitive functioning COVID-19, SARS-CoV-2 cognition, and COVID-19 cognition. Included in literature review were papers with adult participants who had a confirmed diagnosis of COVID-19, journals written in English, and studies which used standardised and objective measures. Self-report measures were not included. IQR = Interquartile Range, SD = standard deviation, Montreal Cognitive Assessment (MoCA), Mini-Mental State Exam (MMSE).

**Finding summary**

Many existing cognitive studies of COVID-19 that used objective testing have not appropriately controlled for demographics, mental health, and comorbid medical conditions. This lack of adjustment probably explains some of the large variance in the observed prevalence of CI. In addition, the previous literature on cognition in recovering COVID-19 patients has focused on patients who were hospitalized with severe acute COVID-19 disease. Such patients can show extensive neuropsychological impairment including focal insults due to stroke. Furthermore, most cognitive studies are cross-sectional, and often have relatively small sample sizes. Finally, studies have tended to consider olfaction and cognition separately and olfaction, in most instances, has been tested subjectively rather than objectively.

**Table S1 References in alphabetical order**

Alemanno, F.*, et al.* COVID-19 cognitive deficits after respiratory assistance in the subacute phase: A COVID-rehabilitation unit experience. *PLoS One* 16, e0246590 (2021).

Almeria, M., Cejudo, J.C., Sotoca, J., Deus, J. & Krupinski, J. Cognitive profile following COVID-19 infection: Clinical predictors leading to neuropsychological impairment. *Brain Behav Immun Health* 9, 100163 (2020).

Amalakanti, S., Arepalli, K.V.R. & Jillella, J.P. Cognitive assessment in asymptomatic COVID-19 subjects. *Virusdisease* **32**, 146-149 (2021).

Beaud, V.*, et al.* Pattern of cognitive deficits in severe COVID-19. *J Neurol Neurosurg Psychiatry* **92**, 567-568 (2021).

Becker, J.T.*, et al.* Concurrent validity of a computer-based cognitive screening tool for use in adults with HIV disease. *AIDS Patient Care STDS* **25**, 351-357 (2011).

Bungenberg, J.*, et al.* Long COVID-19: Objectifying most self-reported neurological symptoms. *Ann Clin Transl Neurol* **9**, 141-154 (2022).

De Lorenzo, R.*, et al.* Residual clinical damage after COVID-19: A retrospective and prospective observational cohort study. *PLoS One* **15**, e0239570 (2020).

Del Brutto, O.H.*, et al.* Cognitive decline among individuals with history of mild symptomatic SARS-CoV-2 infection: A longitudinal prospective study nested to a population cohort. *Eur J Neurol* **28**, 3245-3253 (2021).

Del Brutto, O.H., Rumbea, D.A., Recalde, B.Y. & Mera, R.M. Cognitive sequelae of long COVID may not be permanent: A prospective study. *Eur J Neurol* **29**, 1218-1221 (2022).

Frontera, J.A.*, et al.* Trajectories of Neurologic Recovery 12 Months After Hospitalization for COVID-19: A Prospective Longitudinal Study. *Neurology* (2022).

Hampshire, A.*, et al.* Cognitive deficits in people who have recovered from COVID-19. *EClinicalMedicine* **39**, 101044 (2021).

Hellgren, L.*, et al.* Brain MRI and neuropsychological findings at long-term follow-up after COVID-19 hospitalisation: an observational cohort study. *BMJ Open* **11**, e055164 (2021).

Jaywant, A., Vanderlind, W.M., Boas, S.J. & Dickerman, A.L. Behavioral interventions in acute COVID-19 recovery: A new opportunity for integrated care. *Gen Hosp Psychiatry* **69**, 113-114 (2021).

Liu, Y.H.*, et al.* One-Year Trajectory of Cognitive Changes in Older Survivors of COVID-19 in Wuhan, China: A Longitudinal Cohort Study. *JAMA Neurol* (2022).

Mattioli, F.*, et al.* Neurological and cognitive sequelae of Covid-19: a four-month follow-up. *J Neurol* **268**, 4422-4428 (2021).

Mattioli, F.*, et al.* Neurologic and cognitive sequelae after SARS-CoV2 infection: Different impairment for ICU patients. *J Neurol Sci* **432**, 120061 (2022).

Mazza, M.G.*, et al.* Persistent psychopathology and neurocognitive impairment in COVID-19 survivors: Effect of inflammatory biomarkers at three-month follow-up. *Brain Behav Immun* **94**, 138-147 (2021).

Miskowiak, K.W.*, et al.* Cognitive impairments four months after COVID-19 hospital discharge: Pattern, severity, and association with illness variables. *Eur Neuropsychopharmacol* **46**, 39-48 (2021).

Raman, B.*, et al.* Medium-term effects of SARS-CoV-2 infection on multiple vital organs, exercise capacity, cognition, quality of life and mental health, post-hospital discharge. *EClinicalMedicine* **31**, 100683 (2021).

Soldati, A.B.*, et al.* Telephone Screening of Cognitive Status (TICS) in severe COVID-19 patients: Utility in the era of social isolation. *eNeurologicalSci* **22**, 100322 (2021).

Tomasoni, D.*, et al.* Anxiety and depression symptoms after virological clearance of COVID-19: A cross-sectional study in Milan, Italy. *J Med Virol* **93**, 1175-1179 (2021).

Woo, M.S.*, et al.* Frequent neurocognitive deficits after recovery from mild COVID-19. *Brain Commun* **2**, fcaa205 (2020).

Zhou, H.*, et al.* The landscape of cognitive function in recovered COVID-19 patients. *J Psychiatr Res* **129**, 98-102 (2020).

**STROBE statement: Reporting guidelines checklist for cohort, case-control and cross-sectional studies**

| **SECTION** | **ITEM NUMBER** | **CHECKLIST ITEM** | **REPORTED ON PAGE NUMBER:** |
| --- | --- | --- | --- |
| **TITLE AND ABSTRACT** |  |  |  |
|  | 1a | Indicate the study’s design with a commonly used term in the title or the abstract | 2 (implied due char/word limit) |
|  | 1b | Provide in the abstract an informative and balanced summary of what was done and what was found | 2 (implied due char/word limit) |
| **INTRODUCTION** |  |  |  |
| Background and objectives | 2 | Explain the scientific background and rationale for the investigation being reported | 3-4 |
|  | 3 | State specific objectives, including any pre-specified hypotheses | 3-4 |
| **METHODS** |  |  |  |
| Study design | 4 | Present key elements of study design early in the paper | 5 & Fig 1: flow chart |
| Setting | 5 | Describe the setting, locations, and relevant dates, including periods of recruitment, exposure, follow-up, and data collection | 5 |
| Participants | 6a | Cohort study—Give the eligibility criteria, and the sources and methods of selection of participants. Describe methods of follow-up  Case-control study—Give the eligibility criteria, and the sources and methods of case ascertainment and control selection. Give the rationale for the choice of cases and controls  Cross-sectional study—Give the eligibility criteria, and the sources and methods of selection of participants | 5 |
|  | 6b | Cohort study—For matched studies, give matching criteria and number of exposed and unexposed  Case-control study—For matched studies, give matching criteria and the number of controls per case  Variables | 5 |
| Variables | 7 | Clearly define all outcomes, exposures, predictors, potential confounders, and effect modifiers. Give diagnostic criteria, if applicable | 5-7 & sup material |
| Data sources/measurements | 8* | For each variable of interest, give sources of data and details of methods of assessment (measurement). Describe comparability of assessment methods if there is more than one group. | 5-7 & sup material |
| Bias | 9 | Describe any efforts to address potential sources of bias. | 5-9 & sup material |
| Study size | 10 | Explain how the study size was arrived at | 5-9 & sup material |
| Quantitative variables | 11 | Explain how quantitative variables were handled in the analyses. If applicable, describe which groupings were chosen and why . | 5-9 & sup material |
| Statistical methods | 12a | Describe all statistical methods, including those used to control for confounding | 7-10 & sup material |
|  | 12b | Describe any methods used to examine subgroups and interactions | 7-10 & sup material |
|  | 12c | Explain how missing data were addressed | 7-10 & sup material |
|  | 12d | Cohort study—If applicable, explain how loss to follow-up was addressed  Case-control study—If applicable, explain how matching of cases and controls was addressed  Cross-sectional study—If applicable, describe analytical methods taking account of sampling strategy | 7-10 & sup material |
|  | 12e | Describe any sensitivity analyses | 7-10 & sup material |
| **RESULTS** |  |  |  |
| Participants | 13a | Report numbers of individuals at each stage of study—eg numbers potentially eligible, examined for eligibility, confirmed eligible, included in the study, completing follow-up, and analysed | 11 & Table 1 and Table S1) |
|  | 13b | Give reasons for non-participation at each stage | Fig 1: Flow chart & sup material |
|  | 13c | Consider use of a flow diagram | Fig 1: Flow chart |
| Descriptive Data | 14a | Give characteristics of study participants (eg demographic, clinical, social) and information on exposures and potential confounders | Table 1  11 (Table S1) |
|  | 14b | Indicate number of participants with missing data for each variable of interest | Table 1, & sup material |
|  | 14c | Cohort study—Summarise follow-up time (eg, average and total amount) | Tables  Fig 1: Flow chart |
| Outcome Data | 15* | Cohort study—Report numbers of outcome events or summary measures over time  Case-control study—Report numbers in each exposure category, or summary measures of exposure  Cross-sectional study—Report numbers of outcome events or summary measures | Tables  11-14  & sup material |
| Main Results | 16a | Give unadjusted estimates and, if applicable, confounder-adjusted estimates and their precision (e.g. 95% confidence interval). Make clear which confounders were adjusted for and why they were included | Tables  Figures  11-14  & sup material |
|  | 16b | Report category boundaries when continuous variables were categorized | Tables, Figures, & sup material |
|  | 16c | If relevant, consider translating estimates of relative risk into absolute risk for a meaningful time period | n/a |
|  | 16d | Report results of any adjustments for multiple comparisons | 11-14 Tables legends. & sup material |
| Other Analyses | 17a | Report other analyses done—e.g. analyses of subgroups and interactions, and sensitivity analyses | 11-14  Tables and Figures, legends, & material |
|  | 17b | If numerous genetic exposures (genetic variants) were examined, summarize results from all analyses undertaken | n/a |
|  | 17c | If detailed results are available elsewhere, state how they can be accessed | Sup material  Data availability statement |
| **DISCUSSION** |  |  |  |
| Key Results | 18 | Summarise key results with reference to study objectives | 15-21 |
| Limitations | 19 | Discuss limitations of the study, taking into account sources of potential bias or imprecision. Discuss both direction and magnitude of any potential bias | 20-21 |
| Interpretation | 20 | Give a cautious overall interpretation of results considering objectives, limitations, multiplicity of analyses, results from similar studies, and other relevant evidence | 15-21 |
| Generalisability | 21 | Discuss the generalisability (external validity) of the study results  Other information | 15-21 |
| **FUNDING** |  |  |  |
|  | 22 | Give the source of funding and the role of the funders for the present study and, if applicable, for the original study on which the present article is based | Journal website forms |
